# IRES-targeting small molecule inhibits enterovirus 71 replication via allosteric stabilization of a ternary complex

**DOI:** 10.1038/s41467-020-18594-3

**Published:** 2020-09-22

**Authors:** Jesse Davila-Calderon, Neeraj N. Patwardhan, Liang-Yuan Chiu, Andrew Sugarman, Zhengguo Cai, Srinivasa R. Penutmutchu, Mei-Ling Li, Gary Brewer, Amanda E. Hargrove, Blanton S. Tolbert

**Affiliations:** 1 Department of Chemistry, Case Western Reserve University, Cleveland, OH USA; 2 Department of Chemistry, Duke University, Durham, NC USA; 3 Department of Biochemistry and Molecular Biology, Rutgers Robert Wood Johnson Medical School, Piscataway, NJ USA

**Keywords:** RNA, Target identification

## Abstract

Enterovirus 71 (EV71) poses serious threats to human health, particularly in Southeast Asia, and no drugs or vaccines are available. Previous work identified the stem loop II structure of the EV71 internal ribosomal entry site as vital to viral translation and a potential target. After screening an RNA-biased library using a peptide-displacement assay, we identify DMA-135 as a dose-dependent inhibitor of viral translation and replication with no significant toxicity in cell-based studies. Structural, biophysical, and biochemical characterization support an allosteric mechanism in which DMA-135 induces a conformational change in the RNA structure that stabilizes a ternary complex with the AUF1 protein, thus repressing translation. This mechanism is supported by pull-down experiments in cell culture. These detailed studies establish enterovirus RNA structures as promising drug targets while revealing an approach and mechanism of action that should be broadly applicable to functional RNA targeting.

## Abstract (web summary)

Human enterovirus 71 (EV71) contains an internal ribosome entry site (IRES) that promotes translation of viral RNA. Here the authors show that an antiviral small molecule DMA-135 binds to the EV71 IRES RNA, inducing conformational change and stabilizing a ternary complex to repress translation.

## Introduction

Human enterovirus 71 (EV71) is one of the major etiological agents of hand, foot, and mouth disease among children worldwide. Typical EV71 infections manifest with mild flu-like symptoms that can be overcome by the immune system; however, more severe cases can lead to neurological disorders, paralysis, respiratory failure, and even death. EV71 outbreaks in parts of the Asia-Pacific, such as a recent (2018) epidemic in Vietnam where 53,000 children were hospitalized and six died, emphasize the severity of disease progression and the urgency to develop antivirals or an effective vaccine^1^. To that point, the World Health Organization discussed including EV71 and related D68 in its Blueprint List of Priority Diseases^1,2^.

**Fig. 1:**
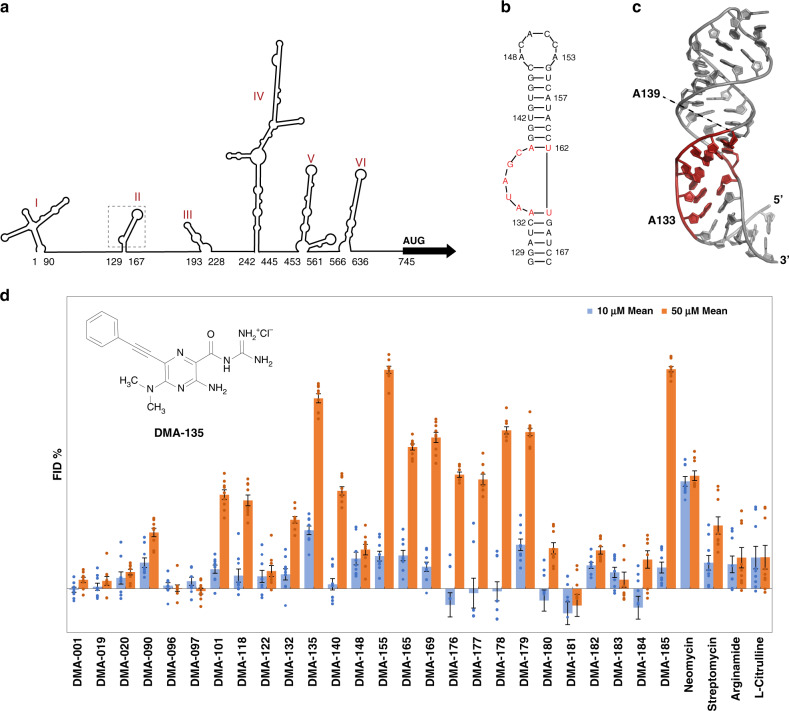
RNA-biased small molecules bind to the EV71 SLII IRES domain. **a** Schematic of the consensus secondary structure of the EV71 5′UTR determined using phylogenetic comparisons. **b** Secondary structure of EV71 SLII as validated by NMR spectroscopy^9^. **c** 3D structure (PDB code 5V16) of SLII refined against NOE-derived distant restraints and RDCs^9^. **d** Screening data for small molecules vs the EV71 SLII (wt) IRES domain at 10 and 50 µm concentrations. Error bars shown are standard errors of mean calculated from three individual replicates. Each replicate data point is the average of nine reads from three parallel readings on three wells (technical replicates). The chemical structure of DMA-135 is shown within the inset. Screening conditions were carried out in 50 mm Tris, 50 mm KCl, 0.01% Triton-X-100, 5% DMSO, pH = 7.4; Tat peptide: 60 nm; RNA: 90 nm; small molecule: 10 or 50 µm; excitation *λ* = 485 nm, emission *λ* = 590 nm; incubation time: 30 min.

EV71 belongs to the *picornaviridae* family, and its genome consists of a positive-sense RNA ~7500 nucleotides (nts) long that function to facilitate protein synthesis and virus replication^3,4^. EV71 regulates these events through several mechanisms, including recruitment of cellular proteins to specific regulatory sites located within the 5′ untranslated region (5′UTR). The 5′UTR is a dual-purpose RNA element that is predicted to fold into six stem loops (SLs) (Fig. 1a). SLI facilitates genome replication, whereas SLs II–VI promote cap-independent translation via a type I internal ribosome entry site (IRES)^4^. The IRES is essential to EV71 replication because it internally recruits the ribosome, which in turn drives synthesis of the complete enteroviral proteome.

Extensive studies of the EV71 IRES have revealed functional contributions of different RNA structures along with the collection of cellular proteins that interact with those structures^5–15^. Of particular importance, the SLII domain interacts specifically with at least four cellular RNA-binding proteins (RBPs) and a viral derived small RNA to modulate translation levels^5,7,9,10,16^. The secondary structure of the EV71 SLII domain folds into a phylogenetically conserved hairpin with a 7-nt apical loop and 5-nt bulge (Fig. 1b)^9^. The three-dimensional structure of SLII shows that the apical loop and bulge form well-defined structural motifs (Fig. 1c)^9^. Functional binding sites for the cellular hnRNP A1 and AUF1 proteins reside within the structured bulge, and these sites undergo a conformational change to form stable protein–RNA complexes^5,9^. HnRNP A1 binds the SLII bulge to stimulate translation, whereas AUF1 competes for the bulge to counteract this stimulatory activity so that the levels of IRES-dependent translation are tuned to meet the replication needs of EV71. Genetic mutations or deletions of bulge residues inhibit EV71 replication, presumably by disrupting the protein–SLII regulatory axis that controls viral translation^9,16^. These collective observations provide strong support that the EV71 SLII domain, particularly, the bulge region, is an attractive target to develop antiviral compounds.

Here, we screen an RNA-focused small molecule (SM) library for binding to the EV71 SLII domain to identify five SM ligands that show specificity to the bulge when confirmed independently by nuclear magnetic resonance (NMR) spectroscopy and isothermal titration calorimetry. One of the five SMs attenuates IRES-dependent translation and displays potent dose-dependent antiviral properties in cell culture. The NMR-derived 3D structure of the SM–SLII complex provides evidence that the SM binds to the bulge to induce a conformational change that fully exposes binding sites for AUF1 but not hnRNP A1. Calorimetric titrations of AUF1 into increasing molar ratios of the SM–SLII complex further validate that the SM increases the binding affinity of a ternary complex in a dose-dependent manner. This thermodynamic property of the SM–SLII–AUF1 complex is also independently observed both in an in vitro biochemical assay and in cell culture. When considered together, these results suggest that the SM acts through an allosteric mechanism to inhibit EV71 replication by stabilizing the translation repressive SM–SLII–AUF1 complex and shifting the SLII regulatory axis. Of note, the allosteric mechanism of action of the IRES targeting SM observed here for EV71 is fundamentally distinct from other IRES targeting antiviral SMs. In the case of the HCV IRES, benzimidazole inhibitors bind to the IRES subdomain IIa to induce a conformational change that leads to undocking of the IIb subdomain from the ribosome^17–20^. In the case of the FMDV IRES, a closely related benzimidazole was found to increase flexibility of SLs within domain 3. Thus, this study reports a unique mechanism of action of a SM that targets an IRES domain, and it provides a promising pathway to develop RNA-focused SM inhibitors of EV71 and likely the related EVD68.

## Results

### Identification of SM DMA-135 as an EV71 SLII ligand

To identify ligands for the EV71 SLII domain, a panel of RNA-targeted SMs was screened against the native EV71 SLII domain using a fluorescent indicator displacement (FID) assay. As previously described, this assay utilizes a fluorescently labeled, highly basic peptide fragment as an indicator that can be displaced from a wide range of RNA targets to assess SM binding^21^. We observed tight binding of the indicator peptide to SLII (24.5 ± 4.7 nm—Supplementary Fig. 1), which facilitated the development of a high-throughput FID protocol to screen SMs against low concentrations of SLII (*Z* score 0.49, Supplementary Note 2). The SM library largely contained derivatives of amiloride, previously identified as a tunable RNA-binding scaffold^22,23^ (Supplementary Fig. 2), along with other known RNA-binding SMs. The screening assay was performed at two different SM concentrations to identify both strong and weak binding derivatives, and “hits” were defined as causing fluorescent changes >20%. (Fig. 1d). Although 13 SM hits are observed at the higher (50 µm) concentration, only two prominent hits are observed at 10 µm: DMA-135, a C6 phenylacetylene substituted amiloride derivative, (Fig. 1d) and a promiscuous RNA binder, neomycin. Quantitative binding analysis was then performed for five amilorides showing both significant displacement at 50 µm and structural diversity (Fig. 1d and Supplementary Fig. 3). This analysis confirmed that DMA-135 was the tightest SLII binder (CD_50_ = 12 µm, Supplementary Table 1). Although peptide assays for each RNA vary slightly, DMA-135 had been previously shown to have comparable affinity to HIV-1-TAR and HIV-2-TAR, much weaker binding to a model A-site and RRE-IIB RNAs^22^, and no interference from tRNA or DNA. We note that a direct measurement of *K*_d_ via ITC (see below) revealed a tighter binding constant (*K*_d_ ~ 500 nm) for EV71 SLII RNA. To further assess the nature of the highest affinity amiloride-SLII binders, NMR and calorimetric titrations were performed.

### Biophysical characterization of EV71 SLII–SM interactions

**Fig. 2:**
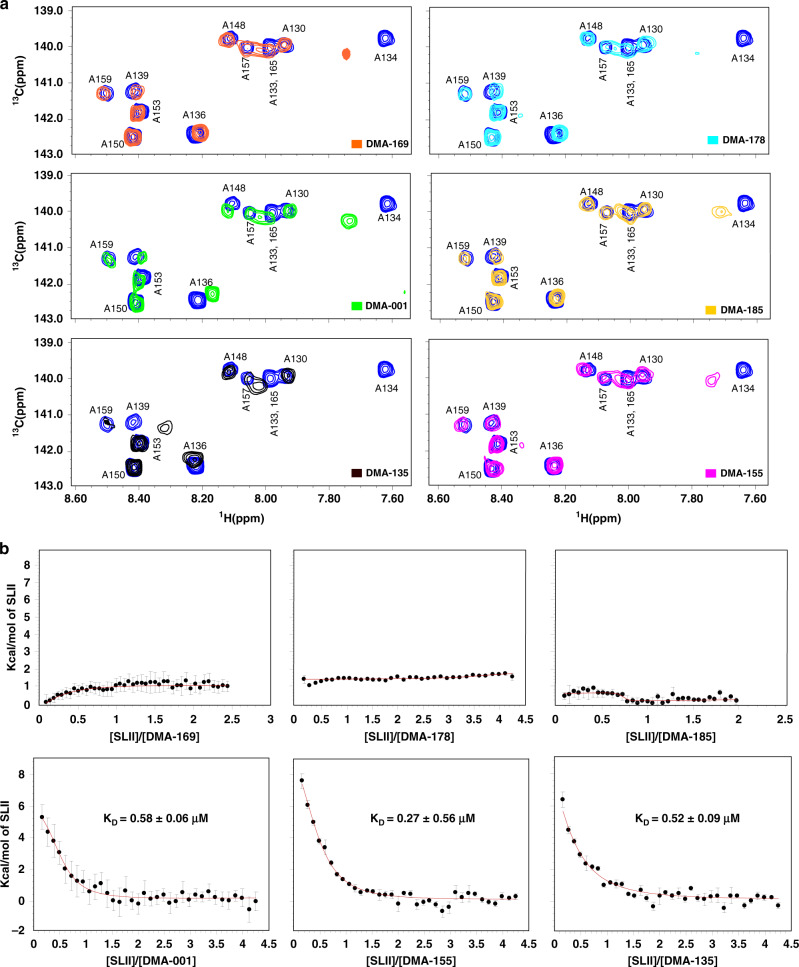
DMAs bind to the EV71 SLII bulge via an entropically driven mechanism. **a** Single-point ^1^H-^13^C TROSY HSQC titrations of A(^13^C)-selectively labeled SLII constructs with the respective DMAs added at fivefold excess. The blue correlation peaks across each spectrum correspond to that of free SLII. The spectra were collected at 900 MHz in 10 mm K_2_HPO_4_, 20 mm KCl, 0.5 mm EDTA, and 4 mm BME (pH 6.5) D_2_O buffer at 298 K. **b** Calorimetric titrations of DMA-001, DMA-135, and DMA-155 reveal a single transition on the binding isotherm characterized by an entropically favored and enthalpically disfavored molecular recognition event. All titration data were processed and analyzed using Affinimeter^25^. The processed thermograms were fit to a 1:1 stoichiometric binding model, represented by the red lines. Reported values for dissociation constants (*K*_D_) and corresponding standard deviation are from triplicate experiments. Goodness of fit (*χ*^2^) of the experimental data to the 1:1 binding model are reported for each titration. The experiments were performed in 10 mm K_2_HPO_4_, 20 mm KCl, 0.5 mm EDTA, and 4 mm DTT (pH 6.5) buffer at 298 K. Data represented as mean ±SD of *n* = 3 experimental replicates. Uncertainties in individual binding isotherms are automatically calculated using Affinimeter and are determined by considering the noise and quality of raw data and the dispersion of the integrated signal as a function of concentration.

We next used NMR titrations to locate the specific structural motifs on SLII that are recognized by the top five SM hits identified from the FID screen along with DMA-001, the core unsubstituted scaffold, as a control based on previous work^22,24^. For these experiments, we prepared an A(^13^C)-selectively labeled SLII construct^9^ and performed single-point ^13^C-^1^H TROSY HSQC titrations. Figure 2a summarizes the effects that the addition of excess (5:1) SMs have on the chemical shifts for each adenosine C8–H8 signal. SLII contains 11 adenosines of which four are located within internal Watson–Crick base pairs (Fig. 1b). A133 and A139 are also involved in Watson–Crick base pairs; however, these residues are adjacent to the 5-nt bulge. None of the adenosines involved in internal base pairs (A130, A157, A159, and A165) or those located in the 7-nt apical loop (A148, A150, A153), show major chemical shift perturbations (CSPs) upon the addition of excess SMs (Fig. 2a). By comparison, all six SMs caused significant CSPs to adenosines adjacent to (A133 and A139) and located within (A134 and A136) the bulge. Thus, these data clearly localize the SLII-binding sites for the SMs to the surface formed by the bulge, in line with previous preferences for amiloride derivatives^22,23^ (Fig. 1b).

To understand the thermodynamics of the SM–SLII interactions, we performed calorimetric titrations. The data reveal that only three (DMA-001, DMA-135, and DMA-155) of the six SMs register a calorimetric response, and those three bind SLII with a favorable change in entropy and an unfavorable change in enthalpy (Fig. 2b). Fits to single-site binding models using Affinimeter^25^ showed excellent agreement with the experimental data therefore allowing reliable determination of the apparent binding constants, which ranged from ~300 to 600 nm (Fig. 2b). Interestingly, the SM DMA-135 binds to SLII with moderately high affinity (*K*_D_ = 520 ± 90 nm), yet it induces the most significant chemical shift perturbations observed in the HSQC titrations (Fig. 2a). These changes include the disappearance of the C8–H8 correlation peak belonging to A134, a large upfield shift of the H8 signal that belongs to A139, and other minor CSPs at residues A133, A136, and A165. By comparison, DMA-001 and DMA-155 induced smaller CSPs with the most notable difference at A134 where the addition of both SMs causes a downfield shift of the C8–H8 correlation peaks (Fig. 2a). In sum, the results indicate that all six SMs interact with SLII through its bulge surface, the binding energies for three of the six SMs derive from large-favorable changes in entropy, and DMA-135 induces the largest changes to the SLII structure as determined by NMR titrations.

### DMA-135 attenuates EV71 translation and replication

**Fig. 3:**
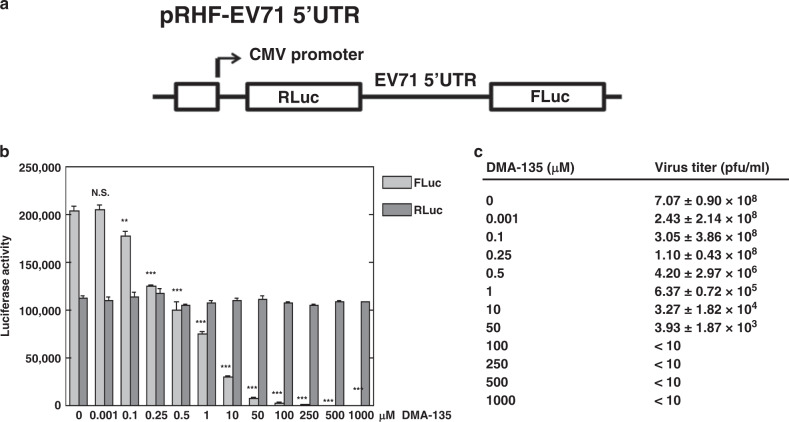
DMA-135 inhibits EV71 IRES-dependent translation and replication. **a** The diagram depicts the bicistronic reporter plasmid used to synthesize RNA for transfections and dual-luciferase assays. **b** Effect of DMA-135 on EV71 IRES activity. SF268 cells were transfected with RLuc-EV71-5′UTR-FLuc RNA and cultured with various concentrations of DMA-135. Luciferase activity was measured 2 days later. Mean values ± standard deviations from three independent experiments (*N* = 3) are shown in the bar graphs. *P* values were determined by unpaired two-tailed Student’s *t* test. ***P < 0.001; **P < 0.01; N.S., not significant. 0.001 µm: *P* = 0.867669; 0.1 µm: *P* = 0.006658; 0.25 µm: *P* = 0.000011; 0.5 µm: *P* = 0.000305; 1 µm: *P* = 0.000031; 10 µm: *P* < 0.00001; 50 µm: *P* < 0.00001; 100 µm: *P* < 0.00001; 250 µm: *P* < 0.00001; 500 µm: *P* < 0.00001; 1000 µm: *P* < 0.00001. **c** Effect of DMA-135 on EV71 replication. SF268 cells were infected with EV71 at an moi = 1. Various concentrations of DMA-135 were added to the cells. Media were harvested 24 hr post infection and assayed for infectious virus by plaque formation in Vero cells. Mean values ± standard deviations from three independent experiments (*N* = 3) are shown. *P* values were determined by unpaired two-tailed Student’s *t* test. 0.001 µm: *P* = 0.025719; 0.1 µm: *P* = 0.153792 (N.S.); 0.25 µm: *P* = 0.000485; 0.5 µm: *P* = 0.000171; 1 µm: *P* = 0.000167; 10 µm: *P* = 0.000167; 50 µm: *P* = 0.000167.

Building on the specificity of the SM–SLII interactions, we next evaluated whether the SMs affected EV71 IRES-dependent translation in cells using a dual-luciferase assay. The description of the bicistronic reporter plasmid, pRHF-EV71-5′UTR, used here has been described elsewhere^13^. In brief, the pRHF-EV71-5′UTR plasmid was used as a template to in vitro transcribe the dual-luciferase reporter RNA (Fig. 3a), which was subsequently transfected into SF268 cells. Each SM was added to the cells at concentrations in the range of 0.001–1000 µm, and after 2 days the activity of Renilla (RLuc) and Firefly (FLuc) were measured. Only DMA-135 showed activity in the assay, and it attenuated IRES-dependent translation (FLuc) in a dose-dependent manner, without having any significant effects on cap-dependent (RLuc) translation (Fig. 3b). At 0.1 μm DMA-135, FLuc activity declined by ~10% relative to the control. FLuc activity declined by 50% at 0.5 μm DMA-135 (*P* = 0.000305) and no Firefly luciferase activity was detected at 100 μm DMA-135, even though cap-dependent (RLuc) translation remained consistent at all concentrations (Fig. 3b).

We next determined whether DMA-135 had antiviral activity by infecting SF268 cells with EV71 at a multiplicity of infection (moi) of 1, followed by the addition of DMA-135. EV71 titers in harvested media were quantitated by plaque formation with Vero cells. DMA-135 inhibits EV71 replication in a dose-dependent manner with a 2-log reduction in viral titers observed at 0.5 μm DMA-135 (Fig. 3c, *P* = 0.000171) and a 5-log reduction when the concentration was increased to 50 μm **(***P* = 0.000167). The IC_50_ of DMA-135 is 7.54 ± 0.0024 μm and the CC_50_ in SF268 and Vero cells is >100 μm. Thus, the collective results indicate that DMA-135 inhibits EV71 replication by attenuating IRES-dependent translation at dosages of relatively low cellular toxicity.

### Solution structure of the SLII-(DMA-135) complex

**Fig. 4:**
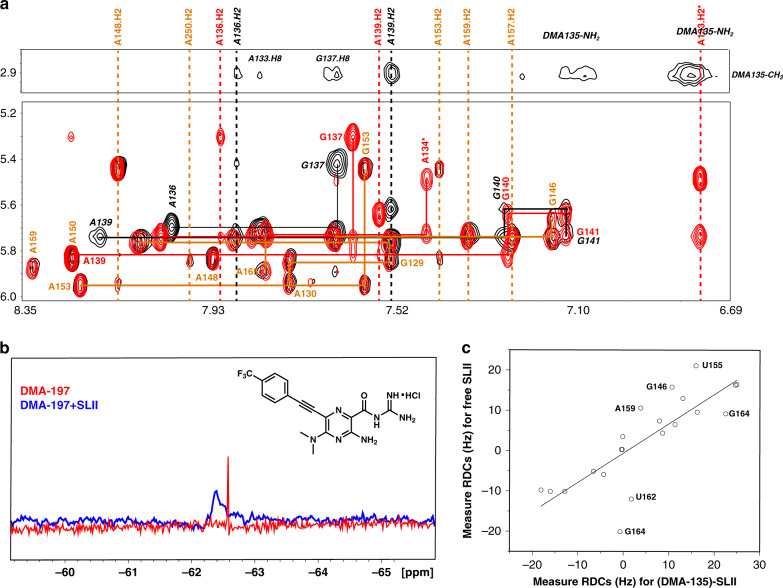
DMA-135 changes the local and global structure of the EV71 SLII IRES domain. **a** Overlay of ^1^H-^1^H NOESY spectra (*t*_m_ = 200 ms) of free SLII (red) and the (DMA-135)-SLII complex (black). The orange lines (solid and dashed) indicate NOE spin systems and chemical shifts that are identical between free SLII and the (DMA-135)-SLII complex. The black lines (solid and dashed) indicate NOE spin systems and chemical shifts of SLII within the complex that are significantly different from those of free SLII (indicated by red solid and dashed lines). Selected region of the ^1^H-^1^H NOESY spectrum of the (DMA-135)-SLII complex showing intermolecular and intramolecular NOEs between methyl protons on DMA-135 and nucleobases of SLII. **b** 1D ^19^F NMR spectra of DMA-197 in the absence (red) and presence of SLII at 1:1 complex (blue). **c** Correlation plot showing agreement between measured RDCs of free SLII and SLII within the DMA-135 complex. All NMR spectra were collected at 900 MHz in 10 mm K_2_HPO_4_, 20 mm KCl, 0.5 mm EDTA, and 4 mm BME (pH 6.5) D_2_O buffer at 298 K.

To better understand the mechanism of action of DMA-135, we solved the NMR solution structure of the SLII-(DMA-135) complex. We previously reported the high-resolution 3D structure of free SLII (Fig. 1c), which was determined using a conjoined NMR-SAXS approach^9^. Informed by the collection of NMR data acquired there, we prepared a CU(^2^H),AG(^2^H_3′–5′′_)-selectively labeled SLII construct to which DMA-135 was added at a 4:1 molar ratio. ^1^H-^1^H NOESY spectra collected on the SLII-(DMA-135) complex with this labeling scheme afforded detection of key intra-NOEs between A and G residues of SLII and inter-NOEs between the label sites on SLII and DMA-135. Figure 4 shows an overlay of the ^1^H-^1^H NOESY (*t*_m_ = 200 ms) spectra of free and the DMA-135 bound form of SLII. Comparisons of the NOESY spectra revealed interesting structural features of the SLII-(DMA-135) complex. First NOEs that involved the A133H2, A133H8, and A134H8 spin systems observed in free SLII were completely missing or highly attenuated in the complex (Fig. 4a). This implies that DMA-135 induces a local change in the structure of SLII that abrogates base stacking at positions 133–134. Second, the intra-NOE interactions that connected A136 to G137 and A139 to G140 were still present in the complex, albeit the chemical shifts of the corresponding H8 and H1’ signals were significantly different from those observed in free SLII. This indicates that the local structure at positions 136–137 and 139–140 are still preserved within the complex but that their chemical environments are different. Third, inter-NOEs between the methyl protons of DMA-135 and A133H8, A136H2, G137H8, and A139H2 firmly establish that DMA-135 forms site-specific contacts with the SLII bulge surface (Fig. 4a). As further validation of the DMA-135 complex, we synthesized a tri-fluorinated analog of DMA-135 (DMA-197) where a trifluoromethyl group was added to allow detection of the complex by ^19^F NMR. Figure 4b provides additional evidence of the SLII-(DMA-135) complex since the sharp 1D ^19^F NMR signal of DMA-197 shifts and becomes severely broaden when bound to SLII. We further verified that DMA-197 indeed binds the bulge surface on SLII by performing a single-point ^13^C-^1^H TROSY HSQC titration as described above for the other DMAs (Supplementary Fig. 4).

**Fig. 5:**
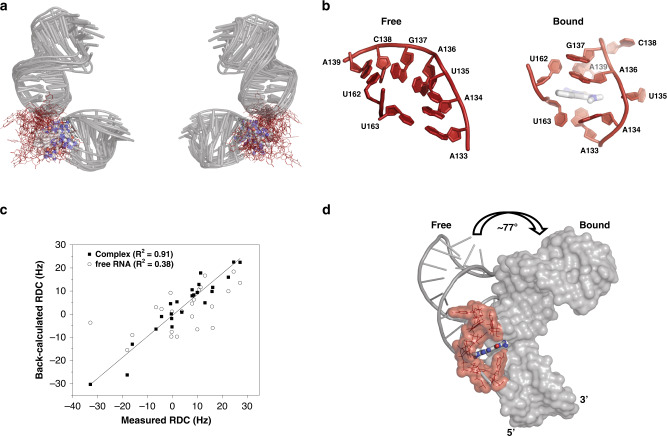
DMA-135 changes the local and global structure of SLII. **a** Superimposition of 10 lowest energy SLII-(DMA-135) complex structures refined in AMBER using NOE-derived distance restraints and RDCs. The overall RMSD for the stably base paired regions is 2.4 Å. **b** Comparison of the bulge stacking interactions of (left) free SLII to that of the SLII-(DMA-135) complex (right). **c** Correlation plot between measured and back-calculated RDCs from a representative low energy SLII-(DMA-135) structure and to back-calculated RDCs from the free SLII structure. **d** Superimposition of the lower helices of free and bound SLII reveals that DMA-135 induces a global change to the SLII structure.

We proceeded to measure ^1^H-^13^C residual dipolar couplings (RDCs) of SLII in complex with DMA-135 so as to determine whether DMA-135 also changes the global structure of SLII. Figure 4c shows that the correlation of measured RDCs between the free and bound forms of SLII are poor, indicating that DMA-135 also changes the global structure of SLII. We then calculated NMR structures of the SLII-(DMA-135) complex using NOEs and RDCs (Table 1). Full details of the structure refinement routine are described in the “Methods” section. The ensemble of SLII-(DMA-135) structures reveal the same overall stereochemical features. First, DMA-135 binds through the bulge of SLII and induces a change in the local stacking patterns at positions 133–135 such that the bases are unstacked and dynamic within the context of the complex (Fig. 5a, b). Binding of DMA-135 to SLII also disrupts a Watson–Crick base pair formed between residues A133 and U163 (Fig. 5b). Second, A136 remains stacked on G137 as observed in free SLII and A139 also gives NOE patterns consistent with its remaining base paired to U162 (Fig. 5b). Because we were limited by inter-NOEs, the conformation of DMA-135 is dynamic within the ensemble so we cannot confidently describe specific interactions to SLII (Fig. 5a). Nevertheless, we observed the appearance of new and broad NOEs within 6.7–7.1 ppm region of the SLII-(DMA-135) NOESY spectrum (Fig. 4a), consistent with the amino group of DMA-135 forming stable hydrogen bonds within the context of the complex.

**Table 1:** NMR and refinement statistics of the (DMA-135)-SLII complex.

	SLII^2231^-DMA-135
**NMR distance and dihedral constraints**	
*Distance restraints*	
Total NOE	506
Intra-residue	162
Inter-residue	344
Sequential (|*i* – *j* | = 1)	245
Nonsequential (|*i* – *j* | > 1)	6
Hydrogen bonds	75
C^H^ RDCs (base pair only)	23
Q_free_ (%)	
Intermolecular (SLII to DMA-135)	29
* Total dihedral angle restraints*	
Nucleic acid	
Base pair	N/A
Sugar pucker	155
Backbone	N/A
Based on A-form geometry	
**Structure statistics**	
*Violations (mean and s.d.)*	
Distance constraints (>0.4 Å)	1.30 ± 1.41
Dihedral angle constraints (>5°)	N/A
Max. dihedral angle violation (>5°)	N/A
Max. distance constraint violation (Å)	0.40 ± 0.38
*Deviations from idealized geometry*	
Bond lengths (Å)	0.01112 ± 0.0007
Bond angles (°)	2.495 ± 0.018
Impropers (°)	N/A
*Average pairwise r.m.s. deviation*^*a*^ *(Å)*	
RNA	
Heavy	3.26 ± 1.92
Backbone^b^	3.25 ± 2.08
Upper helix (12–20 & 27–35)	
Lower helix (1–5 & 35–41)	
*Complex*	0.56 ± 0.11
All complex heavy (C, N, O, P)^c^	3.54 ± 1.86

^a^Statistics were calculated and averaged over an ensemble of the 10 lowest energy structures.

^b^Pairwise r.m.s deviation for backbone was calculated with alignment of C3’, C4’, C5’, O3’, O5’, and P atoms.

^c^Pairwise r.m.s deviation for complex was aligned with base pair region.

As the SLII-(DMA-135) structure was refined with RDCs collected on the complex, we are able to observe that DMA-135 also induces a global change to the structure of SLII. This is evident by comparing the RDC refined structure of free SLII to that of SLII within the DMA-135 complex (Fig. 5d and Supplementary Movie 1). Figure 5d reveals that DMA-135 changes the interhelical angle between the upper and lower helices of SLII by ~77°. This difference is consistent with the observation that DMA-135 changes the local stacking interactions of the bulge (Fig. 5a) such that this leads to a reorientation of the SLII helices within the complex. The interpretation of these collective observations is that DMA-135 binds to the bulge structure to induce a change in its stacking interactions, which exposes A133, A134, and U135 (Fig. 5b). By inducing the exposure of these residues, DMA-135 further changes the interhelical geometry of SLII (Fig. 5d and Supplementary Movie 1).

### DMA-135 stabilizes the AUF1-SLII complex

**Fig. 6:**
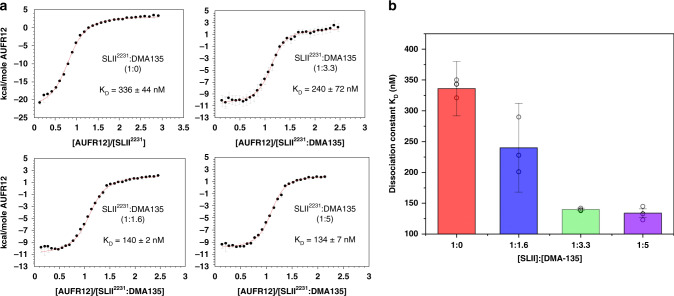
DMA-135 allosterically stabilizes a AUF1-SLII-(DMA-135) ternary complex. **a** Calorimetric titrations of AUF1-RRM1,2 to SLII or to SLII:DMA-135 complex at different molar ratios reveal a single transition in the binding isotherm characterized by an enthalpically driven molecular recognition event. All data were processed and analyzed using Affinimeter^25^. The processed thermograms were fitted to a 1:1 stoichiometric binding model, represented by the red lines. Values for dissociation constants (*K*_D_) and corresponding standard deviation are reported for each titration. The experiments were performed in 10 mm K_2_HPO_4_, 20 mm KCl, 0.5 mm EDTA, and 4 mm BME (pH 6.5) buffer at 298 K. **b** Graphical representation of the dissociation constants for the AUF1-RRM1,2-SLII complex as a function of DMA-135 concentration. Reported *K*_D_ values and corresponding standard deviations are from *n* = 3 experimental replicates. Data represented as mean ± SD of triplicate experiments. Uncertainties in individual binding isotherms are calculated using Affinimeter and are determined by considering the noise and quality of raw data and the dispersion of the integrated signal as a function of concentration.

The cellular proteins, hnRNP A1 and AUF1, bind to the bulge of SLII to stimulate or repress IRES-dependent translation, respectively^5,9,13,16^. We therefore reasoned that DMA-135 might compete either or both of these interactions as part of its mechanism of action. To test this hypothesis, we performed calorimetric titrations using the tandem RNA-binding domains (A1-RRM1,2 and AUF1-RRM1,2) of these proteins and the SLII-(DMA-135) complex prepared at a 5:1 molar ratio. Titrations of A1-RRM1,2 into the SLII-(DMA-135) complex showed that DMA-135 does not impact the binding reaction since the processed thermodynamic parameters are not significantly different when compared with that of the control titration (Supplementary Fig. 5A). In contrast, excess DMA-135 increased the apparent binding affinity of AUF1-RRM1,2 for SLII by approximately threefold (Fig. 6a). We further showed that this property is dose-dependent since the binding dissociation constant decreases as the molar ratio of (DMA-135):SLII increases (Fig. 6b). This property is also specific to DMA-135 because we did not observe significant differences in binding affinities of AUF1-RRM1,2 for SLII when SLII is pre-equilibrated with excess (fivefold) DMA-001 or DMA-155 (Fig. 6a and Supplementary Fig. 4B). Together, these experiments clearly demonstrate that the effects of DMA-135 on SLII-protein binding are specific to AUF1-RRM1,2 in a dose-dependent manner.

Using an ^15^N-labeled AUF1-RRM1,2 construct, we verified by NMR spectroscopy that DMA-135 does not interact with isolated AUF1-RRM1,2 since the ^15^N-^1^H HSQC peaks are identical to those collected without excess DMA-135 (Supplementary Fig. 6A). Instead, we observed the appearance of a new set of correlation peaks when the SLII-(DMA-135) complex is titrated into ^15^N-labeled AUF1-RRM1,2 (Supplementary Fig. 6A). These correlation peaks are not observed in the spectrum recorded when only SLII is titrated into AUF1-RRM1,2 (Supplementary Fig. 6A). When considered together, the calorimetric titrations and NMR data provide evidence for the formation of a (AUF1-RRM1,2)-SLII-(DMA-135) ternary complex (Supplementary Fig. 6B).

**Fig. 7:**
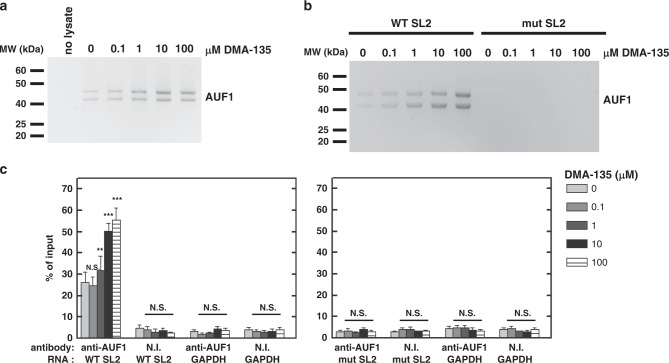
Allosteric effects of DMA-135 within the cellular environment. **a** Protein-biotinylated RNA pull-down experiments were performed to examine the effect of DMA-135 on the interaction between AUF1 and the EV71 SLII IRES domain. Biotinylated SLII was incubated with SF268 cell lysate to which was added the indicated concentrations of DMA-135. Streptavidin-linked beads were used to pull-down biotin-labeled SLII and its associated cellular proteins. Samples were analyzed by western blot using anti-AUF1 antibody. The experiment was performed twice yielding similar results. **b** Biotinylated wild-type SLII or the CCC-bulge mutant SLII were transfected into SF268 cells. Cells were cultured with various concentrations of DMA-135. Cell lysates were used for pull-down assays of SLII-associated proteins as described above. AUF1 bound to SLII RNA was detected by western blotting. The experiment was performed twice yielding similar results. **c** RIP assays for AUF1-EV71 Luc RNA interactions in cells. SF268 cells were transfected with RLuc-EV71 5′UTR-FLuc RNA, harboring the wild-type SLII, or with CCC-bulge mutant SLII and cultured with various concentrations of DMA-135. Lysates were prepared and analyzed by ribonucleoprotein immunoprecipitation (RIP) with non-immune (NI) antibody or AUF1 antibody. Both input and immunoprecipitated materials were analyzed by qRT-PCR for EV71 RNA. NI, non-immune antibody. Mean values±standard deviations from three independent experiments (*N* = 3) are shown. *P* values were determined by unpaired two-tailed Student’s *t* test. ***P < 0.001; **P < 0.01; N.S., not significant.

To test whether DMA-135 affects the stability of the AUF1-SLII complex in a biological context, we designed a set of in vitro and in vivo biochemical experiments. In the in vitro assay, biotinylated SLII was incubated with SF268 cell lysate in the presence of various concentrations of DMA-135. Complexes of AUF1-SLII were captured using streptavidin-conjugated beads and the abundance of AUF1 in complexes was determined by western blotting. The levels of AUF1 increase in the pull-downs as the concentration of DMA-135 increases (Fig. 7a), indicating that the addition of DMA-135 promotes binding of AUF1 to SLII as observed independently by calorimetry (Fig. 6a). To investigate the effects of DMA-135 in a cellular context, biotinylated wild-type SLII and a CCC mutant that abrogates AUF1 and hnRNP A1 binding^5,16^ were transfected into SF268 cells. Cell lysates were prepared 24 hr after transfection and the levels of AUF1 captured by streptavidin pull-down were determined by western blotting. This experiment revealed that the addition of DMA-135 increases binding of AUF1 to wild-type SLII but not to the CCC mutant (Fig. 7b), consistent with the interpretation that DMA-135 promotes the formation of a specific ternary complex with the wild-type RNA.

Finally, we performed RNA–protein immunoprecipitations followed by qRT-PCR to assess whether DMA-135 affects the association of AUF1 with the EV71 5′UTR in cells. SF268 cells were transfected with bicistronic reporter RNAs (see Fig. 3a) containing wild-type SLII or the CCC mutant. Cells were cultured with increasing concentrations of DMA-135. Cell lysates were prepared 24 hr post transfection and RNA–protein complexes were immunoprecipitated using non-immune antibody or AUF1-specific antibody. RNA isolated from immunoprecipitates was then analyzed by qRT-PCR. Wild-type EV71 5′UTR was enriched by antibody against AUF1 relative to non-immune antibody, and the addition of DMA-135 increased the levels of wild-type EV71 5′UTR in complex with AUF1 compared with cells not treated with drug (Fig. 7c, left panel, *P* = 0.000597 at the highest drug concentration). The EV71 5′UTR containing the CCC mutation in SLII was not detected at levels above those for controls (non-immune serum and GAPDH mRNA; Fig. 7c, right panel), consistent with the observation that this mutation abrogates AUF1 binding. Western blot analysis of immunoprecipitates confirmed that the AUF1 antibody precipitated AUF1 while the non-immune antibody did not, as expected (Supplementary Fig. 7). Thus, the independently designed in vitro and in vivo biochemical assays validate that DMA-135 functions to allosterically enhance the binding of AUF1 to SLII by forming a specific ternary complex.

## Discussion

Given the abundance of functional RNA structural elements in human disease systems and in pathogens that infect humans, RNA structures represent significant untapped targets in drug discovery. Here, we report a comprehensive study that combined SM screening, biophysics, and virological assays to successfully identify a SM (DMA-135) that targets the IRES domain of EV71 to inhibit its replication in cell culture. Characterization of DMA-135 activity revealed that it acts as an allosteric inhibitor to increase the binding affinity of AUF1 for the SLII IRES domain and suggested that a ternary complex consisting of AUF1-SLII-(DMA-135) (Supplementary Fig. 6B) attenuates IRES-dependent translation. Our results thus support the targeting of RNA structures of relatively moderate complexity with SMs and that the mechanisms of inhibition include allosteric effects.

In this study, we chose to target the type I IRES domain of EV71, which, along with the related D68, poses serious threats to human health, particularly in regions of Southeast Asia, where it also challenges the economy^1,26–29^. To date, there is no broad-spectrum vaccine or antivirals to slow EV71 progression. The type I IRES domain represents an attractive target for therapeutic intervention as it folds into a highly structured RNA that interacts with multiple cellular proteins to regulate protein synthesis; however, detailed structure-function knowledge is lacking for most of these interactions. SLII is an exception as its sequence conservation and its different binding partners have been thoroughly characterized^5–7,10,13,16^. Those studies demonstrated that both the sequence and structure of the SLII bulge surface are essential for EV71 replication, because genetic mutations or deletions to its residues abrogate a network of interactions that modulate viral translation^9,16^. With that knowledge, we reasoned that SMs might also disrupt the SLII regulatory axis.

The structure of the SLII-(DMA-135) complex and the thermodynamic description of its formation revealed that DMA-135 binds to the shape created by the bulge surface and induces a change that makes a subset of the residues more dynamic (Fig. 5). The residues that transition from a stacked to an unstacked conformation include A133, A134, and U135. These residues match part of the high affinity consensus sequence motif (AU-rich elements) recognized by AUF1^30–32^. The combination of structural changes and increased binding affinity of AUF1 to SLII indicate an allosteric mechanism of action of DMA-135. Support for this mechanism was also observed within the context of the cellular environment. Particularly, a dose-dependent increase in binding of AUF1 to the EV71 5′UTR was observed at DMA-135 treatment concentrations that strongly inhibit viral translation and viral replication.

An additional feature of DMA-135 was that it changed the global architecture of SLII such that the lower and upper helices shift their relative orientations within the complex (Fig. 5d). As we do not know how the SLII structure fits within the larger IRES or how the IRES coordinates ribosome assembly, we cannot confidently assign a function to the relative helical orientation adopted due to base stacking within the bulge. Nevertheless, we previously showed that mutating the central 5′-UAG-3′ bulge sequence to 5′-CCC-3′ also changes the relative helical orientation and this mutant inhibits EV71 replication by attenuating translation^9^. Therefore, this might be a secondary aspect of the mechanism of action of DMA-135.

Although there are reports demonstrating binding of SMs to the HCV and FMDV IRES that result in structural changes^17,19,20^, it is important to note that different IRES types possess different structures and mechanisms of action. The type III HCV IRES promotes internal initiation of translation by direct interaction with the 40 S ribosomal subunit. The type II FMDV IRES and type I EV71 IRES act via binding eukaryotic initiation factors and host RBPs. Likewise, the IRES of HCV, FMDV, and EV71 each present distinct structural organization. The EV71 IRES is composed of six SL domains in which numbers II–VI comprise the IRES. The FMDV IRES has four SL domains, 2–5 (or H-L), in which there are tertiary interactions within domain 3 and rearrangements within domain 4 resulting from eIF-4G binding. Finally, the HCV IRES possess four SL domains. Neither HCV nor FMDV possess a structure similar to EV71 SLII^33,34^. Moreover, the SMs that target HCV appear to function by changing how the IRES binds the 40 S subunit^19^, whereas those that target FMDV increase flexibility of domain 3. Taken together, our work presents a mechanism of action by which DMA-135 functions to allosterically recruit AUF1, which in turn downregulates EV71 translation

In sum, we report a thorough study of a SM–RNA complex that inhibits EV71 replication by attenuating translation. Through the process, we also discovered that the SM acts as an allosteric inhibitor through the formation of a ternary SM–RNA-protein complex. We also note that, although amiloride derivatives have been shown to interact modestly with other RNAs such as HIV-1-TAR, DMA-135 has not been shown to have biological activity prior to this work, and that the combination of unique SM-induced RNA structural changes that lead to the allosteric recruitment of AUF1; specific biological activity of DMA-135; and its lack of toxicity in these studies reflect a high level of functional selectivity. This assessment is further supported by the close agreement between the measured binding affinities and biological IC_50_ values. We believe that this study serves as strong evidence to support drug discovery efforts intended to target RNA structures while revealing promising approaches and potential mechanisms of action. Along those lines, ongoing efforts include optimizing the binding affinity of DMA-135 and also evaluating its utility as a broad-spectrum inhibitor against the related EVD68, which also contains a phylogenetically conserved SLII domain.

## Methods

### RNA preparation

EV71 SL II was prepared by in vitro transcription using recombinant T7 RNA polymerase that was overexpressed and purified from BL21 (DE3) cells. Synthetic DNA templates, corresponding to the EV71 2231 isolate, were purchased from Integrated DNA Technologies (Coralville, IA). Transcription reactions were performed using standard procedures and consisted of 3–6 mL reaction volumes containing unlabeled rNTPs, (C^13^/N^15^)-labeled rNTPs or ^2^H-labeled rNTPs wherein positions 3′–5′′ and H5 are deuterated. Following synthesis, samples were purified to homogeneity by 10% denaturing polyacrylamide gel electrophoresis (PAGE), excised from the gel, electroeluted, and desalted via exhaustive washing of the samples with RNase-free water using a Millipore Amicon Ultra-4 centrifugal filter device. Samples were annealed by heating at 95 °C for 2 minutes and flash-cooled on ice. Samples were subsequently concentrated to desired NMR levels using a Millipore Amicon Ultra-4 centrifugal filter device, followed by addition of buffer salts only (5 mm K_2_HPO_4_ (pH 6.5)) or with buffer salts plus the addition of 0.5 mm ethylenediaminetetraacetic acid (EDTA), 20 mm KCl, and 4 mm beta-mercaptoethanol (BME). Concentration of samples was determined using the RNA theoretical molar extinction coefficient, and NMR samples ranged from 0.05 to 0.2 mm at 200 μL. RNA for all titration experiments was annealed as described above. Post annealing, all RNAs were washed into their respective buffers (see below), using a Millipore Amicon Ultra-4 centrifugal filter device.

### SM synthesis and library preparation

Synthesis of amiloride derivatives DMA-001, DMA-019, DMA-096, DMA-097, DMA-101, DMA-132, DMA-135, DMA-155, DMA-165, DMA-169, DMA-178, and DMA-185 have been previously reported^22,23^. 4-Trifluoromethyl derivative DMA-197 was synthesized via the previously reported two step procedure using Sonogashira coupling reaction followed by guanidinylation^22^. Structures of SMs studied along with the characterization data for derivative DMA-197 presented in Supplementary Fig. 2 and Supplementary Note 1. Other SMs studied—neomycin, streptomycin, mitoxantrone, Hoechst 33258, argininamide, and l-citrulline were purchased from commercial suppliers and used as is. All SMs except neomycin and streptomycin were dissolved in dimethyl sulfoxide (DMSO) at a stock concentration of 50 mm and used in assays as described below. Neomycin and streptomycin are insoluble in DMSO and hence dissolved in water at 50 mm concentration.

### Binding assay for indicator peptide to EV71 SLII RNA

Binding constant of the fluorescently labeled peptide for EV71 SLII RNA, was measured using previously reported procedure^21^. In brief, a Tat-derived peptide [N-(5-FAM)-AAARKKRRQRRRAAAK(TAMRA)-C] was purchased from Lifetein and dissolved at a concentration of 120 nm in the assay buffer containing 50 mm Tris, 50 mm KCl, 0.01% Triton-X-100, pH = 7.4. RNA stock solutions were serially diluted in assay buffer from 0 to 2 µm concentrations. The Tat peptide solution (8 µL) and RNA solution (8 µL) were combined in a Corning low volume round-bottom black 384-well plates, shaken on an orbital shaker for 5 min, centrifuged at 3220 × *g* for 1 min, and allowed to incubate for 30 min (Final Tat concentration = 60 nm, final RNA concentrations = 0–1000 nm). The 384-well plates were then read on a Clariostar monochromator (BMG Labtech) microplate reader using the excitation and emission wavelengths of 485 nm (FAM) and 590 nm (TAMRA), respectively. The fluorescence intensities at each RNA concentration were normalized to the Tat-only control wells. Dissociation constants (*K*_d_) were calculated by fitting the observed relative fluorescence intensity values at each concentration to Eq. 1 using the GraphPad Prism curve fitting software. Assays were conducted in triplicates and each replicate contained three technical replicates.

1 $${\mathit{Y}} = {\mathit{Bmax}} * {\mathit{X}}/(K{\mathit{d}} + {\mathrm{X}}) + {\mathit{NS}} * {\mathit{X}} + {\mathrm{Background}}$$

Where, *Y* = total binding; *X* = Conc. of added ligand; *B*max = maximum binding; *K*_d_ = equilibrium dissociation constant; *NS*: slope of nonlinear regression; Background: measured binding without the ligand.

### SM screening using the Tat peptide-displacement assay

Screening of the SM library against the EV71 SLII wt RNA was performed using the Tat peptide-displacement assay previously reported^21^. In brief, Tat peptide was diluted at a concentration of 180 nm in the assay buffer. RNA solution was prepared by diluting the stock solution in the assay buffer at 270 nm concentration. These concentrations were chosen to provide the best fluorescence intensity possible in the bound state. SMs were diluted to concentrations of 30 µm and 150 µm. Tat peptide (6 µL), RNA (6 µL), and SM solution (6 µL) were combined in a 384-well plate. Final concentrations in each assay well were as follows-Tat peptide: 60 nm; RNA conc: 90 nm; SM: 0 µm, 10 µm, and 50 µm. The well-plate was shaken on an orbital shaker for 5 min, centrifuged at 4000 rpm for 1 min, and allowed to incubate in dark for 30 min and read using the excitation and emission wavelengths of 485 nm (FAM) and 590 nm (TAMRA), respectively. Percentage displacement of Tat peptide from RNA was calculated using Eq. 2. Each screening experiment was performed in triplicates and each replicate contains a technical triplicate.

2 $${\mathrm{\% FID}} = 100 - \left( {100 \times \frac{{\mathit{F}}}{{{\mathit{F}}0}}} \right)$$

$$ F = F_{{\mathrm{(ligand + RNA + Tat)}}}- F_{{\mathrm{(RNA + ligand)}}}$$

$${{F}}_{{0}} = F_{{\mathrm{(RNA + Tat)}}}\qquad \,\,\,$$

Molecules that show >20% FID at tested concentrations of 10 µm or 50 µm, were labeled as hits of the screening assay at that concentration.

### Full binding titrations of amiloride derivatives with EV71 SLII

Titrations of amiloride derivatives for binding to both the wild-type and mutant RNAs was performed as previously described^3,21^. In brief, SMs were diluted in the assay buffer to achieve final assay concentrations between 0 and 334 µm. Tat peptide (6 µL), RNA (6 µL), and SM solution (6 µL) were combined in a 384-well plate, shaken on an orbital shaker for 5 min, centrifuged at 4000 rpm for 1 min, and allowed to incubate for 30 min before being read using the excitation and emission wavelengths of 485 nm (FAM) and 590 nm (TAMRA), respectively. Observed fluorescence at each SM concentration was normalized to the fluorescence of Tat peptide: RNA complex. Competitive displacement of 50% of peptide from RNA was calculated by fitting the normalized data to the Eqs. 3 and 4 below using the GraphPad Prism curve fitting software. Assays were conducted in triplicate and each replicate contained three technical replicates. Errors presented are the standard errors of mean from three replicates.

3 $$y = A1 + \frac{(A2 - A1)}{(1 + 10^{(Logx^{0} - x)p})}$$

4 $$CD_{50} = 10^{Logx^{0}} \qquad \qquad \qquad \qquad $$

### Protein purification

The AUF1 protein used in this study (residues from 70 to 239) was subcloned into the pMCSG7 vector^35^; subsequently overexpressed as an N-terminal (His)_6_-tagged fusion protein in BL21(DE3) cells and grown in either LB broth or M9 minimal medium supplemented with ^15^NH_4_CL (0.5 g/L). The (His)_6_-tagged AUF1 construct was purified via nickel affinity chromatography on a Hi-trap column (GE Biosciences) followed by a Hi-trap Q column (GE Biosciences). The (His)_6_-purification tag was cleaved using the tobacco etch virus (TEV) enzyme and the cleavage mixture was then loaded onto Hi-trap columns (GE Biosciences) to isolate the protein of interest. Subsequently, the AUF1 construct was loaded onto a HiLoad 16/600 Superdex 75 pg (GE Bioscience) gel filtration column and eluted into the desired buffer. Protein stock solutions were kept in a buffer consisting of 10 mm K2HPO_4_, 0.5 mm EDTA, 20 mm KCl, and 4 mm BME (pH 6.5). The UP1 protein (A1-RRM1,2) used in this study (residues 1–196) was subcloned into a pET28a vector and subsequently overexpressed as a C-terminal (His)6-tagged fusion protein in BL21 (DE3) cells and grown in LB Broth. The C-terminal (His)6-UP1 was purified via nickel affinity chromatography on Hi-Trap column (GE Bioscience) and subsequently loaded onto a HiLoad 16/600 Superdex 75 pg (GE Bioscience) gel filtration column and eluted into the desired buffer. Protein stock solutions were kept in a buffer consisting of 10 mm K2HPO4, 120 mm NaCl, 0.5 mm EDTA, and 5 mm DTT at pH 6.5. Protein homogeneity was confirmed by SDS-PAGE and concentrations was determined using the theoretical molar extinction coefficient.

### Isothermal titration calorimetry experiments

The RNA and the respective DMA samples were prepared and purified as described above. Calorimetric titrations were performed on a VP-ITC calorimeter (Microcal, LLC) at 25 °C into 10 mm K_2_HPO_4_, 20 mm KCl, 0.5 mm EDTA, and 4 mm BME (pH 6.5) buffer, centrifuged and degassed under vacuum before use. SLII at 40 μm was titrated into ~1.4 mL of 1.5 μm of the respective DMA over a series of 32 injections set at 6 μL each. To minimize the accumulation of experimental error associated with batch-to-batch variation, titrations were performed in triplicate. Data were analyzed using KinITC routines supplied with Affinimeter^25^.

The AUF1 construct used in this study was prepared and purified as described above. Calorimetric titrations were performed on a VP-ITC calorimeter (Microcal, LLC) at 25 °C into 10 mm K_2_HPO_4_, 20 mm KCl, 0.5 mm EDTA, and 4 mm BME (pH 6.5) buffer, centrifuged and degassed under vacuum before use. AUF1 at 100 μm was titrated into ~1.4 mL of 8 μm SLII:DMA-135 complex at the following RNA:DMA ratios: 1:0, 1:1.6, 1:3.3, 1:5. To minimize the accumulation of experimental error associated with batch-to-batch variation, titrations were performed in duplicate. Data were analyzed using KinITC routines supplied with Affinimeter.

### Cells and virus

SF268 (human glioblastoma) cells were grown in Roswell Park Memorial Institute (RPMI) medium supplemented with 10% fetal bovine serum (Gibco) and Vero (African green monkey kidney) cells were grown in MEM medium supplemented with 10% fetal bovine serum (Gibco)^16^. Cells were infected with EV71 (TW/2231/98) at indicated moi (multiplicity of infection) and incubated 1 h at 37 °C for adsorption. Unbound virus was removed, and cells were refed fresh medium with various concentrations of DMA-135. Media from infected cells were harvested 24 h post infection, and virus titers were determined by plaque assay on Vero cells.

### Determination of EV71 IRES activity

The bicistronic reporter plasmid pRHF-EV71-5′UTR, which contains the EV71 IRES between Renilla and Firefly luciferase open reading frames^13^ was used as the template for synthesis of capped reporter RNA using the MAXIscript kit (ThermoFisher). SF268 cells were seeded in 24-well plates. Two hundred ng of reporter RNA, 5 µl of SuperFect (Qiagen), and 400 µl of RPMI with 10% fetal calf serum were combined and added to one well of cells. Cells were incubated at 37 °C for 4 h, medium was changed, and various concentrations of DMA-135 were added. Two days after transfection, IRES activity was determined by measuring Renilla luciferase (RLuc) and Firefly luciferase (FLuc) activities using a dual-luciferase reporter assay system (Promega).

### Plaque reduction assay

Antiviral activity of DMA-135 was determined by plaque reduction assay. In brief, Vero cells were infected with EV71 at a concentration of 10^7^ pfu/ml, which gives ~60 plaques per well. DMA-135 was diluted and incubated in the agar-medium overlay at 37 °C for 3 days. Cells were stained and plaques were counted. The concentration of DMA-135 required to reduce the number of plaques by 50% relative to the virus control was expressed as IC_50._ Experiments were performed in triplicate.

### Cytotoxicity assays

Various concentrations of DMA-135 were added to SF268 and Vero cell lines. The cells were incubated at 37 °C for 96 h. Cell viability was determined by MTT assay and measured at 570 nm according to the manufacturer’s instructions (EMD Millipore). All experiments were performed in triplicate. The concentration of DMA-135 required to reduce cell viability to 50% of the control cells was expressed as CC_50_.

### Pull-down of protein-biotinylated RNA complexes using streptavidin beads

To prepare SF268 cell extracts, SF268 cells were cultured until confluent. Cells were pelleted, washed with cold PBS, resuspended in CHAPS buffer (10 mm Tris-HCl (pH 7.4), 1 mm MgCl_2_, 1 mm EGTA, 0.5% CHAPS, 10% glycerol, 0.1 mm phenylmethylsulfonyl fluoride, 5 mm 2-ME), and incubated on ice for 30 min. Cells were lysed by centrifugation at 10,000 × *g* for 10 min at 4 °C, and the supernatants were collected^5^. Biotin-labeled RLuc-EV71 5′UTR (wild-type or CCC mutant)-FLuc and biotin-labeled SL2 (wild-type or CCC mutant) were synthesized using biotin-16-UTP (Roche). Binding reaction mixtures contained 200 µg of cell extract proteins and 3 µg of biotinylated RNA. The final volumes of reaction mixtures were adjusted to 100 µl with RNA mobility shift buffer (5 mm HEPES (pH 7.1), 40 mm KCl, 0.1 mm EDTA, 2 mm MgCl_2_, 2 mm dithiothreitol, 1 U RNasin, and 0.25 mg/ml heparin). The mixtures were incubated for 15 min at 30 °C and then added to 400 µl of streptavidin MagneSphere Paramagnetic Particles (Promega) for binding at room temperature for 10 min. Beads, containing RNA–protein complexes, were washed with RNA mobility shift buffer lacking heparin. After the last wash, 15 µl of 6× SDS-PAGE sample buffer was added to the beads and the mixtures were incubated for 10 min at room temperature. Binding reactions and pull-downs were carried out^5^. The eluted proteins were fractionated by 10% SDS-PAGE. AUF1 was detected by western blot analyses using anti-AUF1 rabbit polyclonal antibody at 1:15,000 dilution.

### RNP-immunoprecipitation

Immunoprecipitations of endogenous protein–RNA complexes were used to examine association of AUF1 with EV71 5′UTR in SF268 cells. For pre-clearing, lysates of 6 × 10^7^ SF268 cells were incubated with rabbit non-immune serum (Sigma) for 45 min at 4 °C, then magnetic Dynabeads coupled to protein A (Invitrogen) were added for 30 min at 4 °C; beads were removed with a magnet. For immunoprecipitations, fresh beads were coated with anti-AUF1 or rabbit non-immune serum in NT-2 buffer (50 mm Tris-HCl (pH 7.4), 1 mm MgCl_2_, 150 mm NaCl and 0.05% Nonidet P-40) and washed. Pre-cleared cell lysates (1 mg protein) were incubated with 50 µl of coated beads in 200 µl of NT-2 buffer supplemented with 2.5 µl of RNase Out (Invitrogen) and 2 µl of 100 mm DTT for 3.5 hr at 4 °C with constant rocking. Beads were washed eight times with ice-cold NT-2 buffer and two times with NT-2 buffer supplemented with 0.5 m urea. Proteins were digested with proteinase K (Promega), and RNAs were purified by phenol–chloroform extraction and ethanol precipitation^5^. For quantitation of mRNAs in precipitates, purified RNAs were reverse transcribed into cDNAs using the High Capacity cDNA Reverse Transcription kit (Applied Biosystems), followed by SYBR green quantitative PCRs. The forward and reverse primer sequences, respectively, are as follows: EV71_F: 5′-CCCACCCACAGGGCCCACTGG-3′ and EV71_R: 5′-CGTTGATTTACAGCTTCTAA-3′; GAPDH_F: 5′-TTTAACTCTGGTAAAGTGGATATTGTTG-3′ and GAPDH_R 5′-ATTTCCATTGATGACAAGCTTCC-3′.

For one experiment, an aliquot of each reaction was removed prior to addition of proteinase K and RNA purification. These samples were analyzed by western blot to confirm specific immunoprecipitation of AUF1.

### NMR data acquisition

NMR spectra were recorded on Bruker Advance (700, 800, and 900 MHz) high-field spectrometers. ^1^H-^13^C HSQC titrations were collected in 100% D_2_O at 303 K on SLII constructs with selectively labeled ^13^C(rATP). ^1^H-^1^H NOESY (*t*_m_ = 200 ms) spectra of the (DMA-135)-SLII complex were collected in 100% D_2_O at 303 K on SLII samples prepared with selectively deuterated rNTPs, rRTP ^2^H (3′,4′,5′5′′) and rYTP^2^H (5,3′,4′,5′5′′). RDCs were measured using ^1^H-^13^C TROSY HSQC experiments for both isotropic and anisotropic ^13^C-selectively labeled SLII samples. Partial alignment was achieved via the addition of Pf1 filamentous bacteriophage (ASLA) to a concentration of ~15 mg/mL; phage concentration was validated via ^2^H splitting at 900 MHz. RDC values were determined by taking the difference in ^1^J_CH_ coupling under anisotropic and isotropic conditions. ^1^H-^15^N HSQC titrations of either unlabeled SLII, DMA-135 or SLII:DMA-135 complex into ^15^N-labeled AUF1 were performed. Spectra were collected at the following molar ratios SLI^I^:AUF1 (1:1), DMA-135: AUF1 (4:1), SLII:DMA-135: AUF (1:4:1). All NMR data were processed with NMRPipie/NMRDraw^36^ and analyzed using NMRView J^37^ or Sparky^38^.

The amiloride DMA-135 protons were assigned using 1D proton, 1D carbon and NOESY NMR experiments. Spectra were collected at a concentration of 200 μm of the SM dissolved in D_2_O. The dimethylamino group protons were observed at a chemical shift of 3.00 ppm.

### HADDOCK-derived starting models of the (DMA-135)-SLII complex

Since the ^1^H-^1^H NOESY data (Fig. 4) revealed that DMA-135 abrogates base stacking interactions within the SLII bulge, we first performed molecular dynamic simulations on the NMR-refined SLII structure (PDB code = 5V16) using distance restraints with lower bounds set at 7 Å for bulge residues A133-U135. This simulation resulted in an ensemble of SLII structures where the bulge loop was remodeled so as to disrupt local base stacking interactions at residues A133-U135. We next performed HADDOCK^39^ (version 2.2 Guru mode) docking calculations with these remodeled structures and incorporated the chemical shift perturbations as well as the inter-NOEs observed between SLII and DMA-135 as active restraints. The bulge loop (residues A133-C138) was treated as semi-flexible. One thousand rigid body (DMA-135)-SLII complex structures were calculated, and the most favorable structures (*n* = 195) underwent further semi-flexible and water refinement. The output structures were clustered with a minimum size of 4, resulting in seven total clusters. Sixteen representative (DMA-135)-SLII complex structures from the three best-scoring HADDOCK clusters were selected for further refinement in AMBER^40,41^. The molecular operating environment was used to generate the initial structure for DMA-135. Protonated DMA-135 was processed through PRODRG2 server.

### AMBER equilibration and simulation workflow

Sixteen starting (DMA-135)-SLII complex structures were prepared for AMBER using Antechamber and tLEaP. Initial equilibration of these systems was performed through a series of energy minimizations. Final frames of the minimization steps were simulated using pmemd.cuda for 50 ns each in AMBER 16 using the ff99bsc0_OL3_ force field. In both minimization and production, simulations were performed in implicit solvent using generalized Born model (igb = 1) and a salt concentration of 10 mm.

### Details of the Antechamber step

Antechamber and QM calculations were utilized to build input files optimized for simulation in AMBER. The parameters for the DMA-135 were obtained using the GAFF force field and the Antechamber package. DMA-135 structures were prepared for Antechamber and further AMBER calculations through sqm (QM) calculation of atomic point charges and the reduce program. Antechamber was performed on each structural model, assuming a net charge of +1 owing to a protonated guanidinium group. An additional force field containing specific parameters was written for each SM–RNA construct using the parmchk feature of Antechamber. The output files of Antechamber calculations were utilized to perform two rounds of tLEaP. First, GAFF and ff14SB were used to generate AMBER parameter files for DMA-135, and a library (*.lib file) was created. Second, the ff99bsc_OL3_ and ff14SB were sourced and the aforementioned library loaded, allowing the generation of AMBER parameters (*.prmtop and *.inpcrd) for each starting structure of the complex.

### Simulation and refinement of the (DMA-135)-SLII complex structures

Initial (DMA-135)-SLII structures were minimized in sander using 2000 steps of steepest descent followed by 2000 steps of conjugate gradient. A 24 Å cutoff for non-bonded interactions was input alongside a 10 Å cutoff for calculation of the Born radii. After minimization, production MD simulations were performed using GPU-accelerated Particle Mesh Ewald Molecular Dynamics (pmemd) on NIVIDIA Tesla P100 GPUs. During this stage, NOE (20 kcal mol^−1^ Å^−1^) hydrogen bonding and chirality restraints were used. Torsion angle restraints were manually incorporated to maintain the aromatic rings of DMA-135 in a planar orientation. The guanidinium group was assigned to its lowest energy resonance structure. For the 50 ns simulation (total 25,000,000 steps of 2 fs each), a non-bonded cutoff of 999.9 was used alongside a 10-Å cutoff for calculation of the Born radii. SHAKE was used to constrain bonds involving hydrogen and Langevin dynamics with a collision frequency of 2.0 ps^−1^ was used to control temperature. The RNA was heated from 0 to 300 K over 100 ps and simulated for the remainder of the 50-ns simulation at 300 K.

After 50 ns of simulation, cpptraj was used to generate structures from the trajectory files, extracting the final frame of each simulation. (200 per starting structure). The 16 structures were simulated for 1 ns in Amber tools 15 using sander and the ff99bsc0XOL3 force field. At this stage, both minimization and production used implicit solvent (igb = 1), a salt concentration of 10 mm, a non-bonded cutoff of 24 Å and a 10-Å cutoff for calculation of the Born radii. RDCs were incorporated into the refinement simulation with a weak-weighting coefficient of 0.01 as single-value restraints alongside the hydrogen bonding, NOE, and chirality restraints utilized in the previous phase of calculations. Structures were prepared once more in Antechamber and minimized over 4000 steps (2000 steps of steepest descent followed by 2000 steps of conjugate gradient). After this final minimization, the experimentally determined RDC values were fit to the structures by freezing the coordinates of the structure to allow generation of the molecular alignment tensor. In the simulation step, the structures were allowed to move in order to refine them according to available experimental restraints. Temperature scaling was arranged such that the system spent 1 ns at 300 K before cooling to 0 K. Upon completion of this refinement step, the final frames from each of the 16 trajectories were extracted to individual *.pdb file. These final structures were ranked according to their total energies, with the lowest 10 represented here. Back-calculated RDCs were extracted from sander output files and assessed for their agreement with measured values. Once calculation completed, the 10 lowest energy structures for each construct were visualized in PyMOL and checked using MolProbity.

### Reporting summary

Further information on research design is available in the Nature Research Reporting Summary linked to this article.

## Supplementary information

Supplementary Information

Reporting Summary

Description of Additional Supplementary Files

Supplementary Movie 1

## Data Availability

The chemical shifts and the atomic coordinates of the SLII-(DMA-135) complex have been deposited in the Biological Magnetic Resonance Bank (BMRB ID: 50268) and the Protein Data Bank (PDB ID: 6XB7). Source data for the figures and supplementary figures are provided as a Source Data file. All data and materials are available from the authors upon reasonable request. Source data are provided with this paper.

## Source data

Source Data
