# Supplementary material for: IRES-targeting small molecule inhibits enterovirus 71 replication via allosteric stabilization of a ternary complex
Source: Nat Commun. 2020 Sep 22;11:4775. doi: 10.1038/s41467-020-18594-3 (PMC7508794; doi:10.1038/s41467-020-18594-3)

IRES-targeting Small Molecule Inhibits Enterovirus 71 Replication via Allosteric Stabilization of a Ternary Complex

Jesse Davila-Calderon[1], Neeraj Patwardhan[2], Liang-Yuan-Chiu[1], Andrew Sugarman[1], Zhengguo Cai[2], Srinivasa R. Penutmutchu[1], Mei-Ling Li[3], Gary Brewer[3], Amanda E. Hargrove[2*] and Blanton S. Tolbert[1*]*

Department of Chemistry, Case Western Reserve University, Cleveland, OH
Department of Chemistry, Duke University, Durham, NC
Department of Biochemistry and Molecular Biology, Rutgers Robert Wood Johnson Medical School, Piscataway, NJ

*email: blanton.tolbert@case.edu or Amanda.hargrove@duke.edu or brewerga@rwjms.rutgers.edu

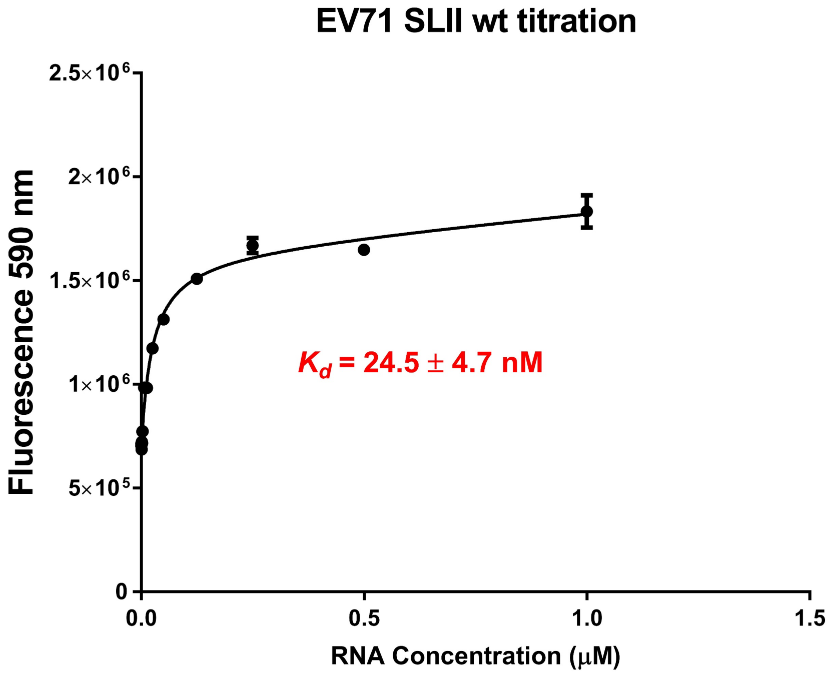

Supplementary Figure 1. Binding assay for Tat peptide to SLII. 60 nM Tat peptide was titrated with EV71 SLII RNA (0~1 μM). Buffer: 50 mM Tris, 50 mM KCl, 0.01% Triton-X-100, 5% DMSO, pH = 7.4. Data are presented as mean values +/- SEM and measured from 3 technical replicates over 3 biological replicates (n=9 for SEM calculation).

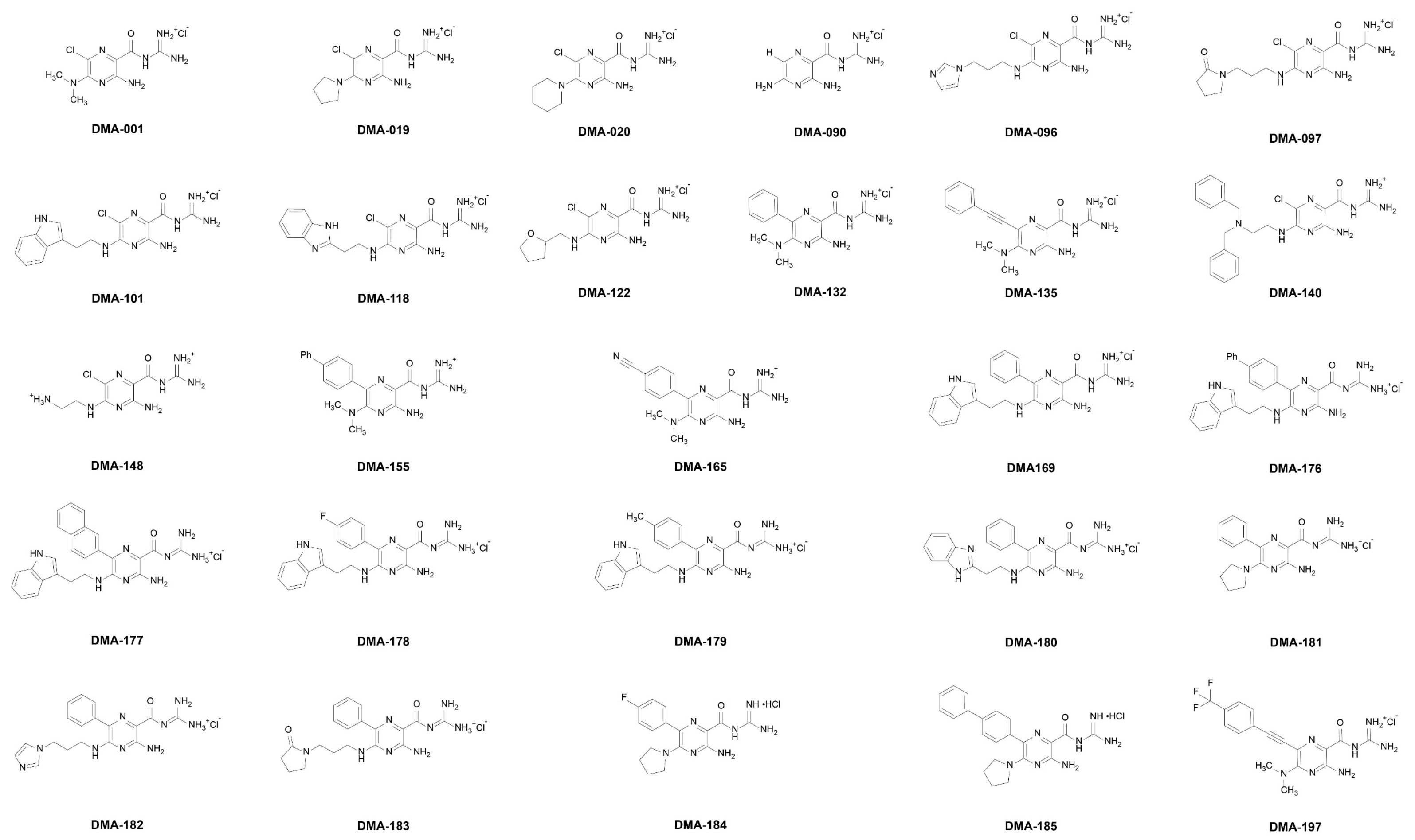

Supplementary Figure 2. Structures of amiloride derivatives utilized in this study

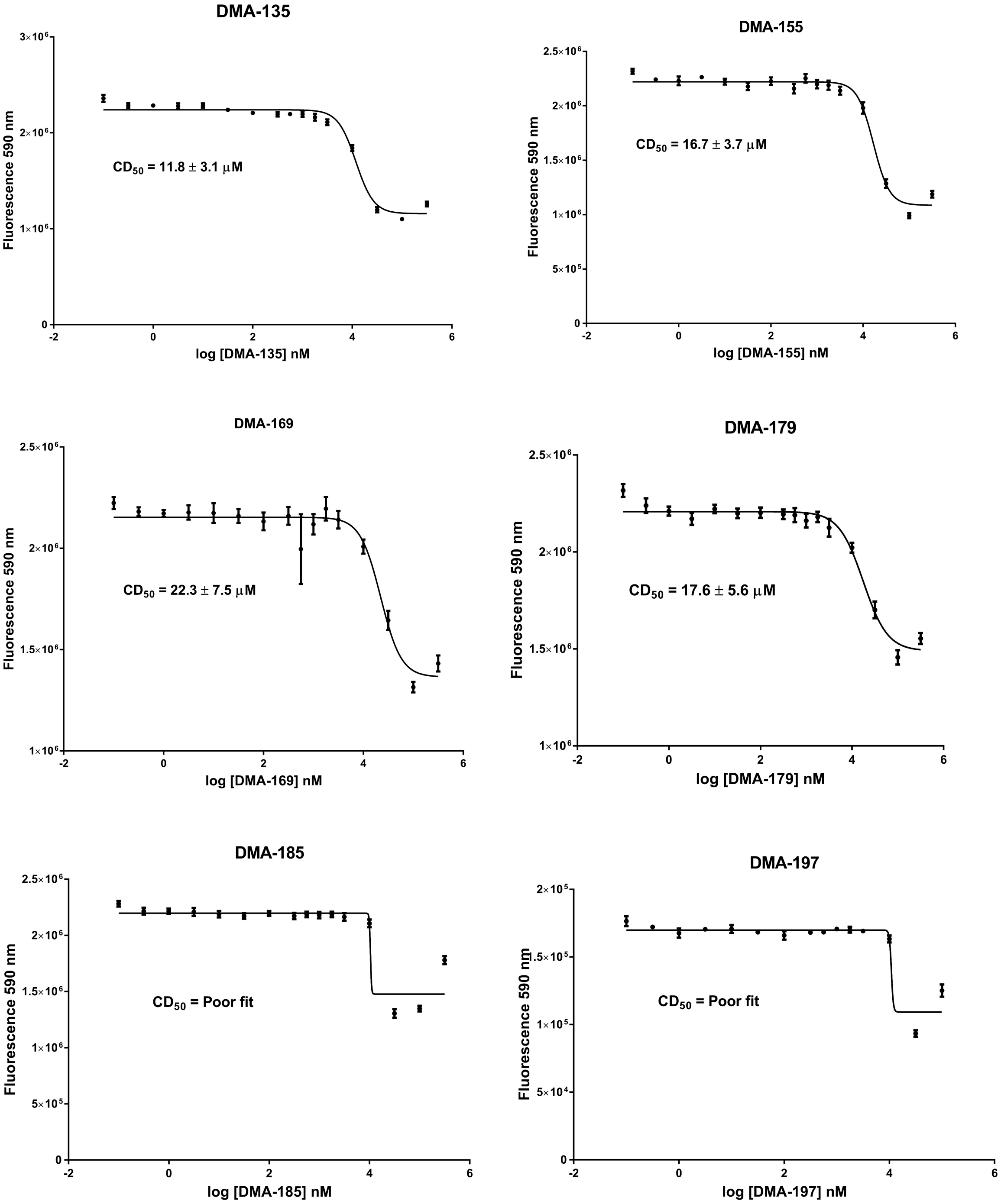

Supplementary Figure 3. Full binding curves of amiloride molecules vs EV71 SLII RNA. [Tat peptide] = 60 nM, [EV71 SLII] = 90 nM, log[DMA]/nM = -1~5.5 (data point was deleted for DMA-197 at highest concentration due to small molecule precipitation), buffer: 50 mM Tris, 50 mM KCl, 0.01% Triton-X-100, 5% DMSO, pH = 7.4. Data are presented as mean values +/- SEM and measured from 3 technical replicates over 3 biological replicates (n=9 for SEM calculation). Specifically, DMA-197 were measured from 3 technical replicates over 2 biological replicates due to data export issue, so N=6 for this compound.

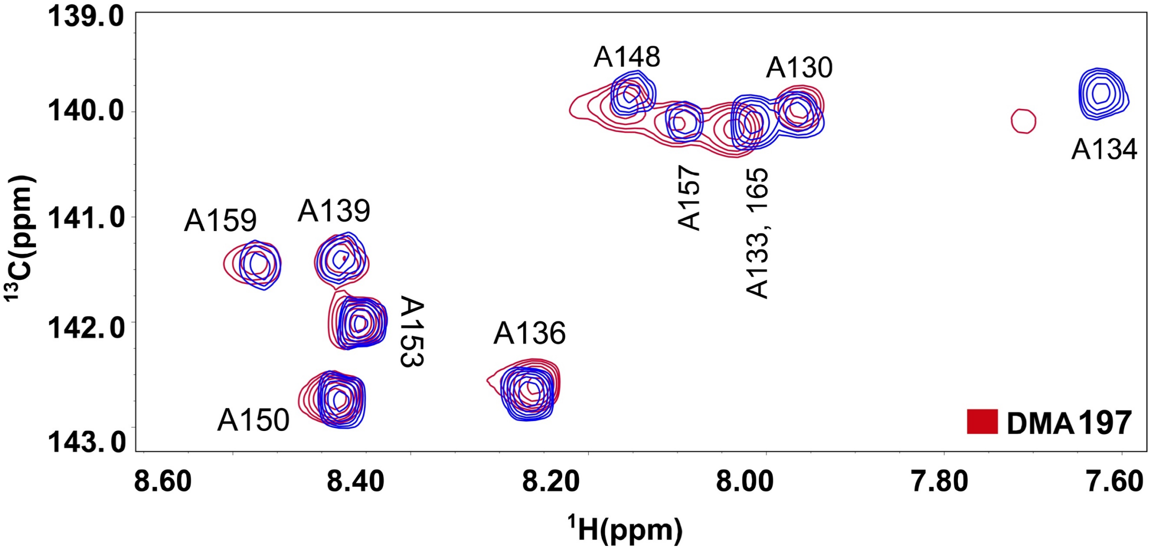

Supplementary Figure 4. Single-point ^{1}H-^{13}C TROSY HSQC titrations of A(^{13}C)-selectively labeled SLII constructs with DMA-197 at 5-fold excess

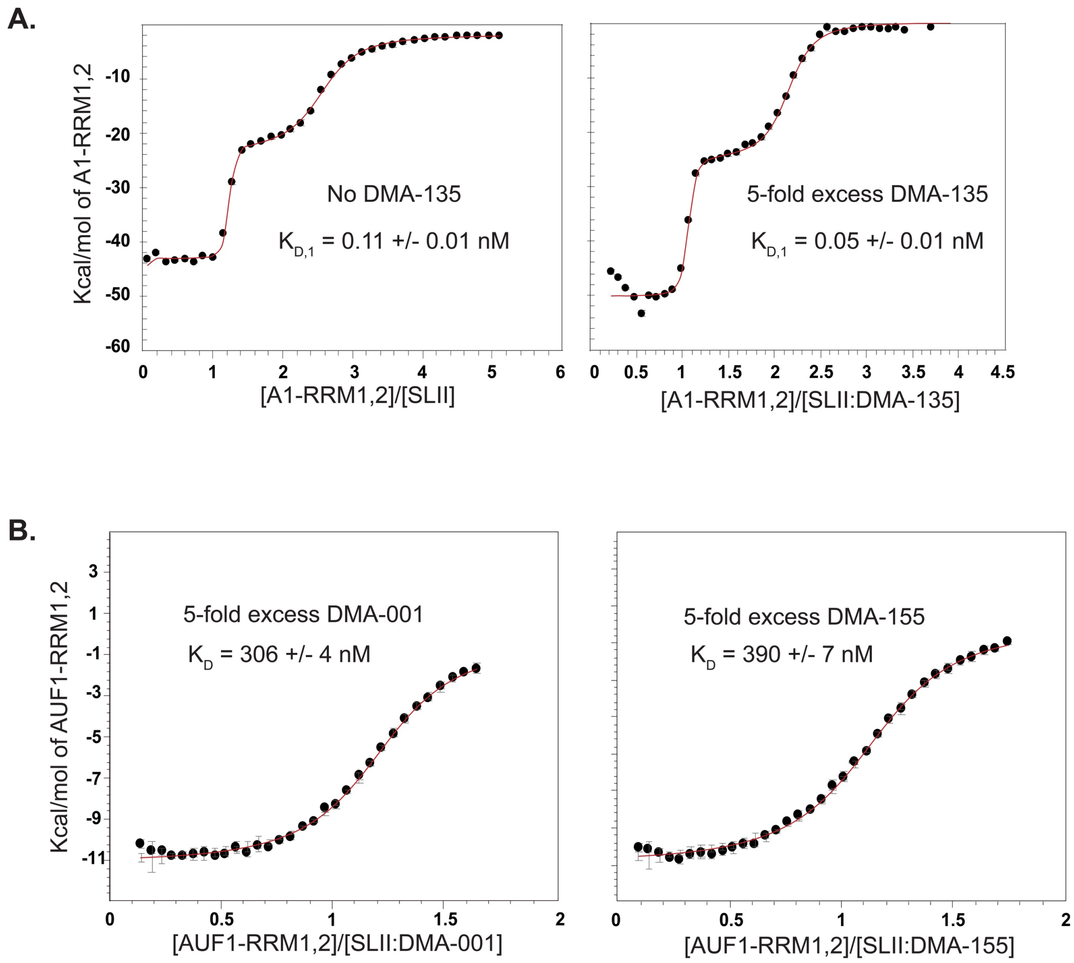

Supplementary Figure 5. (A) Calorimetric titrations of A1-RRM1,2 into free SLII (left) and the SLII-(DMA-135) complex prepared at a 1:5 molar ratio. (B) Calorimetric titrations of AUF1-RRM1,2 into (left) SLII-(DMA-001) and (right) SLII-(DMA-155) each prepared at a 1:5 molar ratio.The experiments were performed in 10 mM K2HPO4, 20 mM KCl, 0.5 mM EDTA, and 4 mMDTT (pH 6.5) buffer at 298 K. Data represented as mean +/- SD of n=2 experimental replicates. Uncertainties in individual binding isotherms are calculated using Affinimeter and are determined by considering the noise and quality of raw data and the dispersion of the integrated signal as a function of concentration.

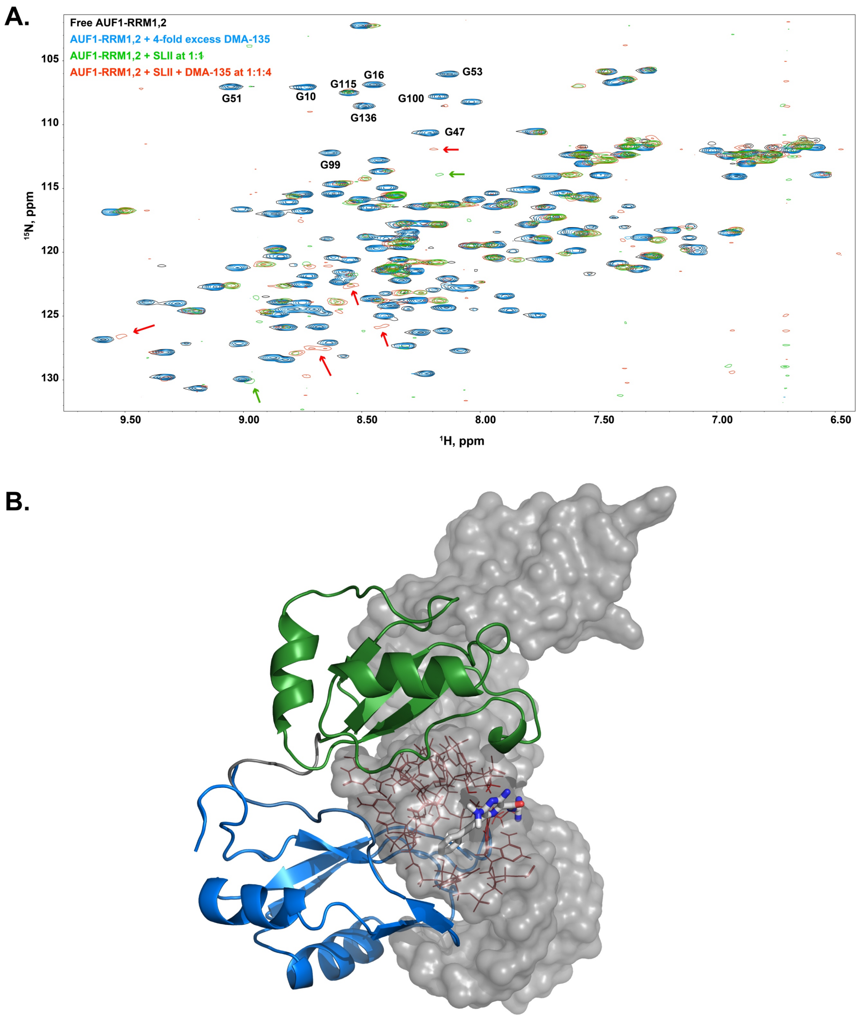

Supplementary Figure 6. (A) Single-point ^{1}H-^{15}N HSQC titrations of ^{15}N-labeled AUF1-RRM1,2 (black) with excess DMA-135 (blue); with SLII added at a 1:1 molar ratio (green); or with the SLII-(DMA-135) complex prepared at a 1:4 molar ratio (red). Red arrows indicate new correlation peaks only observed in the presence of SLII-(DMA-135). (B) Structural model of the AUF1,2-SLII-(DMA-135) ternary complex. The structural model was calculated in HADDOCK using the observed AUF1-RRM1,2 chemical shift perturbations as docking restraints. The structure of AUF1-RRM1,2 is a preliminary solution NMR structure that will described elsewhere.

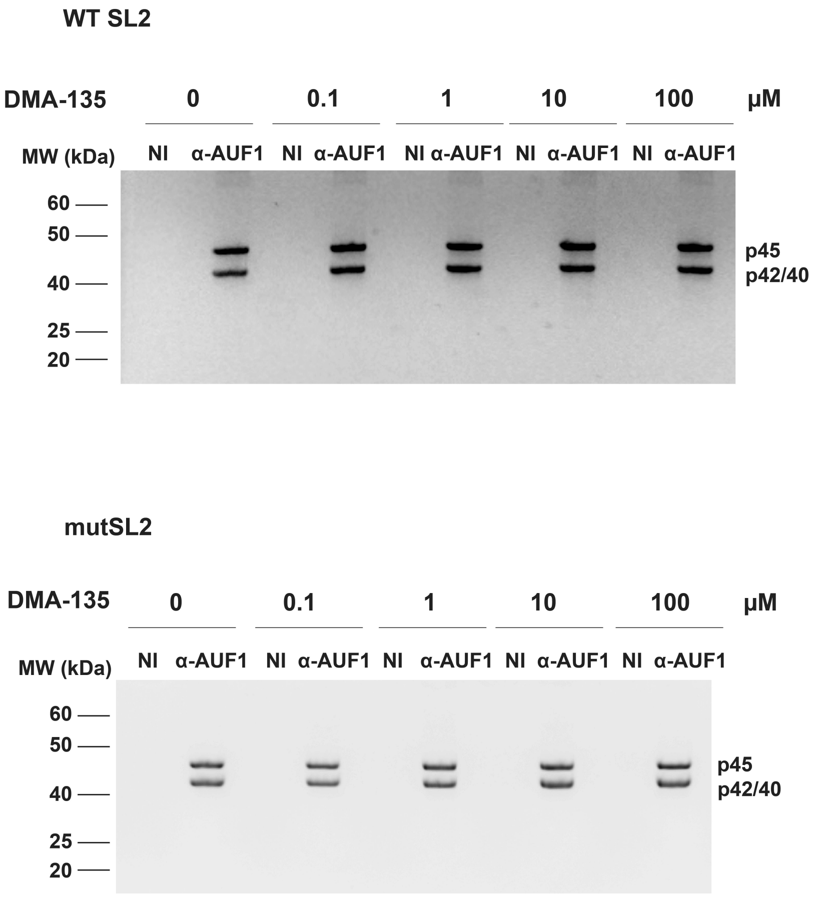

Supplementary Figure 7. Aliquots of samples immunoprecipitated with either anti-AUF1 or non-immune serum (N.I.) from Figure 7C were analyzed by Western blot to verify anti-AUF1–dependent recovery of AUF1. Western blot analysis was performed for one complete RIP experiment since the three replicate experiments yielded similar quantitative RT-PCR results. The specific AUF1 isoforms are indicated. Note that immunoprecipitation using N.I. serum did not precipitate AUF1, as expected. Uncropped blot images are provided as source data.

Supplementary Table 1. CD_{50} values for a selected set of small molecules

**Molecule**	**CD_{50} (µM)**
DMA-135	11.8 ± 3.1
DMA-155	16.7 ± 3.7
DMA-169	22.3 ± 7.5
DMA-178	17.6 ± 5.6
DMA-185	Poor Fit

Supplementary Table 2. Summary of thermodynamic parameters for DMA-SLII and AUF1-SLII-(DMA-135) interactions.

System	ΔG (kcal/mol)	ΔH (kcal/mol)	-TΔS (kcal/mol)	K_D (nM)
**SLII to DMA-001**	-8.6 ± 0.4	12.8 ± 3	-21.4 ± 2	584 ± 62
**SLII to DMA-135**	-8.6 ± 0.1	12 ± 1	-20.7 ± 0.4	525 ± 98
**SLII to DMA-155**	-9.0 ± 0.1	11 ± 5	-20 ± 5	271 ± 59
**SLII to DMA-169**	No Data			
**SLII to DMA-178**	No Data			
**SLII to DMA-185**	No Data			
**AUF1 to SLII**	-8.8 ± 0.1	-25.7 ± 0.7	16.8 ± 0.7	336 ± 44
**AUF1 to SLII: DMA-135 (1:1.6)**	-9.0 ± 0.2	-11.7 ± 0.5	2.7 ± 0.5	240 ± 72
**AUF1 to SLII: DMA-135 (1:3.3)**	-9.34 ± 0.01	-13.91 ± 0.04	4.58 ± 0.04	140 ± 2
**AUF1 to SLII: DMA-135 (1:5)**	-9.37 ± 0.03	-13.5 ± 0.2	4.1 ± 0.2	134 ± 7
**AUF1 to SLII: DMA-001 (1:5)**	-8.88 ± 0.01	-10.9 ± 0.4	2.1 ± 0.4	306 ± 4
**AUF1 to SLII: DMA-155 (1:5)**	-8.73 ± 0.01	-13.11 ± 0.01	4.38 ± 0.02	390 ± 7

Supplementary Note 1. Characterization data for trifluoromethyl derivative DMA-197. Amino(3-amino-5-(dimethylamino)-6-((4-fluorophenyl)ethynyl)pyrazine-2-carboxamido) methaniminium chloride (DMA-197):

^{1}H NMR (500 MHz, Methanol-d4) δ 7.75 – 7.65 (m, 4H), 3.46 – 3.38 (m, 1H, NH), 3.37-3.32 (m, 6H); ^{13}C NMR (126 MHz, MeOD) δ 165.5, 157.1, 155.6, 154.2, 131.3, 131.2, 126.7, 125.3, 112.8, 112.2, 90.2, 89.9, 39.4; HRMS (ESI+): Calculated for $C_{17}H_{17}F_3N_7O$ [M+H]: 392.1441, Found: 392.1448 (± 1.6 ppm). HPLC Analysis: Retention time = 7.106 min, Purity = 95.141%.

^{1}H NMR Spectrum for Amino(3-amino-5-(dimethylamino)-6-((4-fluorophenyl)ethynyl)pyrazine-2-carboxamido) methaniminium chloride (DMA-197):

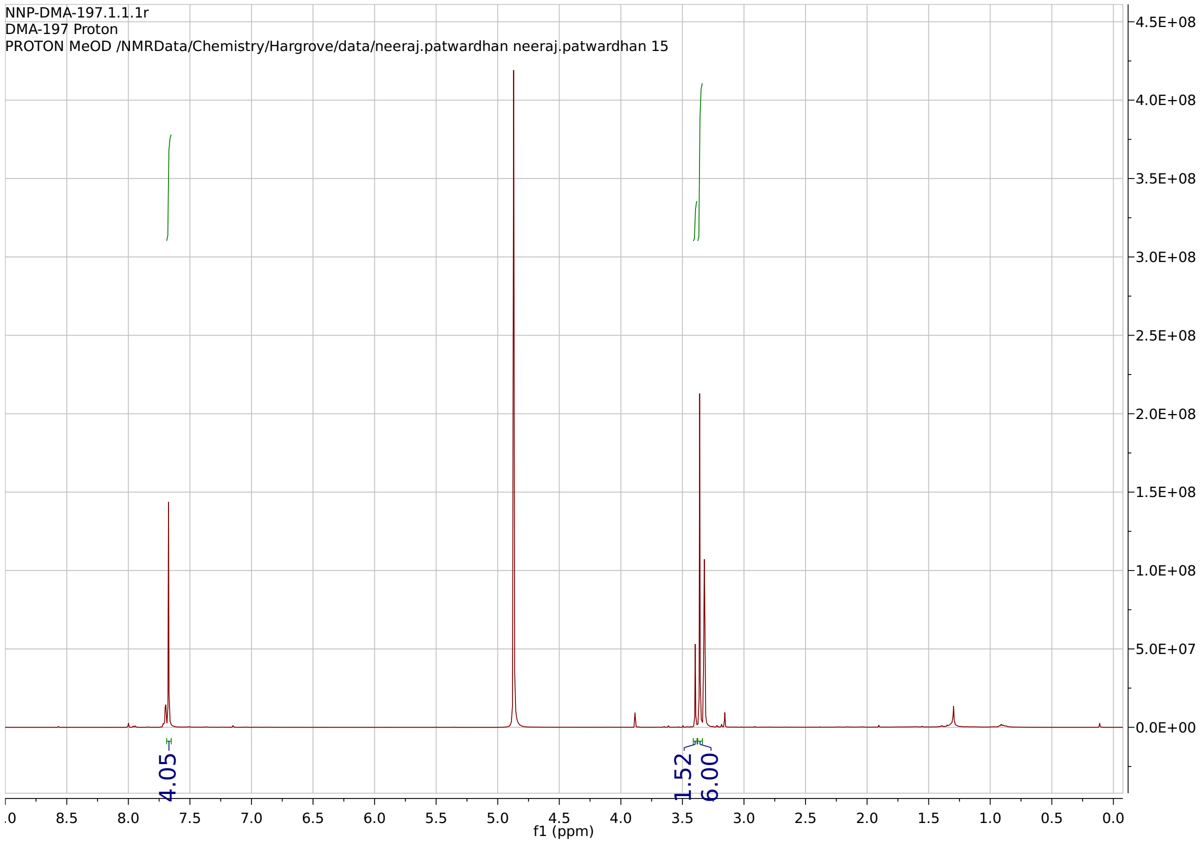

^{13}C NMR Spectrum for Amino(3-amino-5-(dimethylamino)-6-((4-fluorophenyl)ethynyl)pyrazine-2-carboxamido) methaniminium chloride (DMA-197):

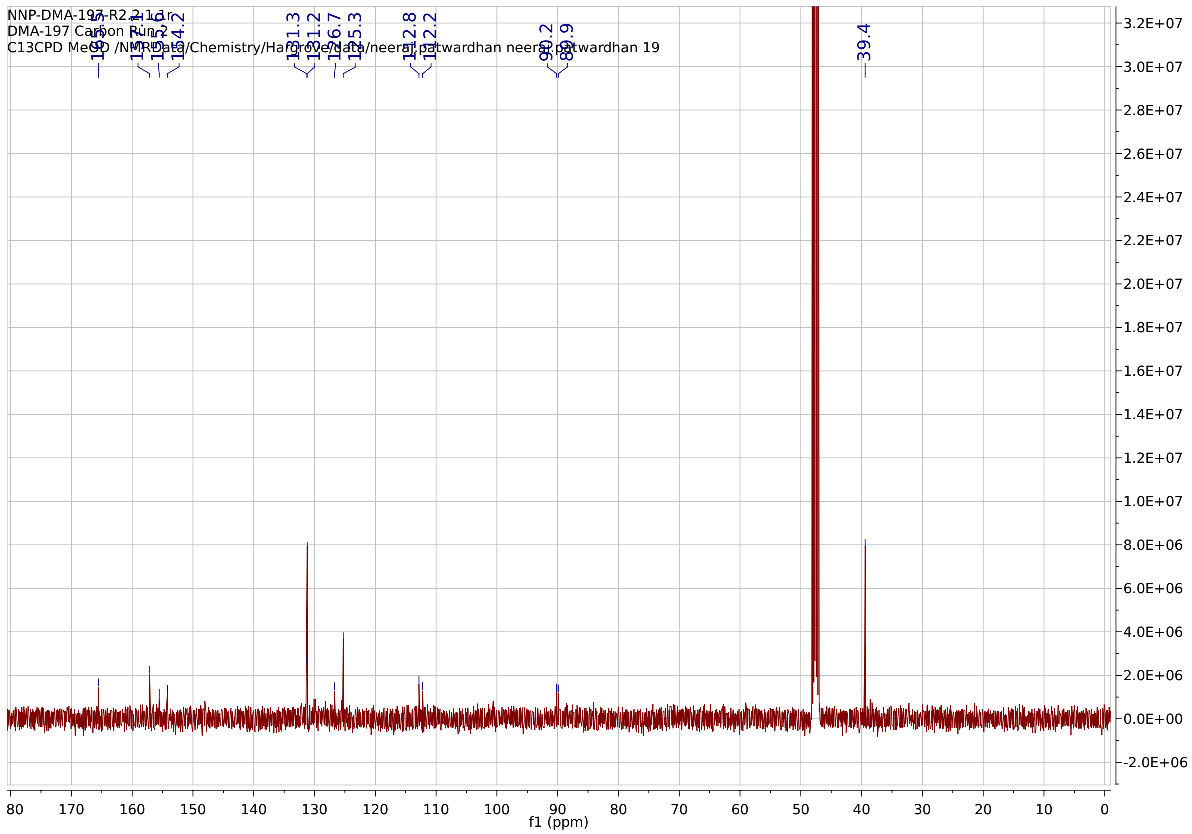

HPLC Chromatogram for for Amino(3-amino-5-(dimethylamino)-6-((4-fluorophenyl)ethynyl)pyrazine-2-carboxamido) methaniminium chloride (DMA-197):

SHIMADZU LabSolutions

Analysis Report

<Sample Information>

Sample Name	: NNPIII-129-R1		
Sample ID	: NNPIII-129-R1		
Data Filename	: NNPIII-129-R1.lcd		
Method Filename	: NNP-Grd10-90_Slow_PDA_D2only.lcm		
Batch Filename	: NNP_04_22_19_R1.lcb		
Vial #	: 1-5	Sample Type	: Unknown
Injection Volume	: 10 uL		
Date Acquired	: 4/22/2019 11:02:53 AM	Acquired by	: chemist
Date Processed	: 4/22/2019 11:25:57 AM	Processed by	: chemist

<Chromatogram>

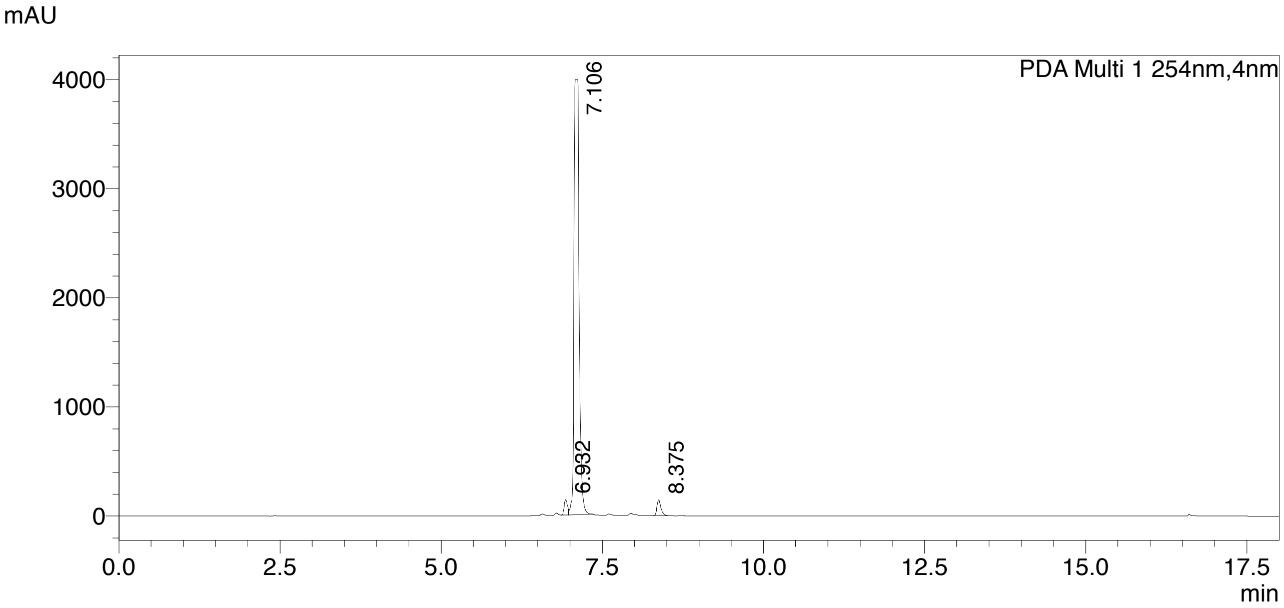

<Peak Table>

PDA Ch1 254nm

Peak#	Ret. Time	Area	Area%
1	6.932	497759	2.160
2	7.106	21925166	95.141
3	8.375	622086	2.699
Total		23045010	100.000

Supplementary Note 2. Small molecule screening using the Tat peptide displacement assay

Z' Scores: Z' Scores for each RNA: peptide system were calculated using the equation below, using 144 data points for each RNA:peptide complex –

$$Z' = 1 - \left(\frac{3\,(\sigma_{(positive)} + \sigma_{(negative)})}{|\,\mu_{(positive)} - \mu_{(negative)}|} \right)$$

$\mu_{(positive)}$ = Mean Fluorescence Intensity for peptide + RNA
$\mu_{(negative)}$ = Mean Fluorescence Intensity for peptide alone
$\sigma_{(positive)}$ = Standard deviation for positive
$\sigma_{(negative)}$ = Standard deviation for negative

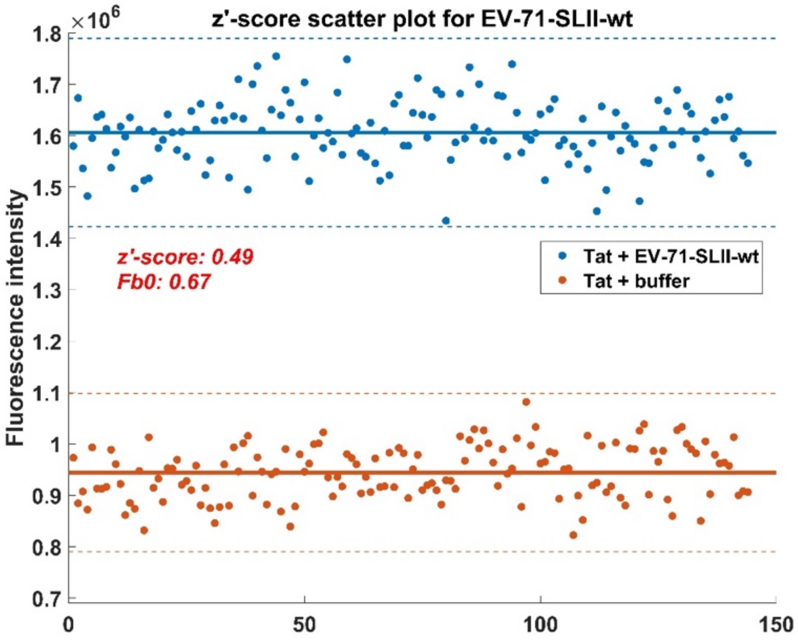

Supplement: Supplementary file 1 — Supplementary Information [file 41467_2020_18594_MOESM1_ESM.pdf]
