# Supplementary material for: IRES-targeting small molecule inhibits enterovirus 71 replication via allosteric stabilization of a ternary complex
Source: Nat Commun. 2020 Sep 22;11:4775. doi: 10.1038/s41467-020-18594-3 (PMC7508794; doi:10.1038/s41467-020-18594-3)
Supplement: Supplementary file 2 — Reporting Summary [file 41467_2020_18594_MOESM2_ESM.pdf]

nature research

Corresponding author(s): Blanton Tolbert, Amanda Hargrove, Gary Brewer

Last updated by author(s): 2020/07/31

# Reporting Summary

Nature Research wishes to improve the reproducibility of the work that we publish. This form provides structure for consistency and transparency in reporting. For further information on Nature Research policies, see Authors & Referees and the Editorial Policy Checklist.

## Statistics

For all statistical analyses, confirm that the following items are present in the figure legend, table legend, main text, or Methods section.

| n/a | Confirmed | |
|---|---|---|
| ☐ | ☒ | The exact sample size (*n*) for each experimental group/condition, given as a discrete number and unit of measurement |
| ☐ | ☒ | A statement on whether measurements were taken from distinct samples or whether the same sample was measured repeatedly |
| ☒ | ☐ | The statistical test(s) used AND whether they are one- or two-sided<br>*Only common tests should be described solely by name; describe more complex techniques in the Methods section.* |
| ☒ | ☐ | A description of all covariates tested |
| ☒ | ☐ | A description of any assumptions or corrections, such as tests of normality and adjustment for multiple comparisons |
| ☐ | ☒ | A full description of the statistical parameters including central tendency (e.g. means) or other basic estimates (e.g. regression coefficient) AND variation (e.g. standard deviation) or associated estimates of uncertainty (e.g. confidence intervals) |
| ☐ | ☒ | For null hypothesis testing, the test statistic (e.g. *F*, *t*, *r*) with confidence intervals, effect sizes, degrees of freedom and *P* value noted<br>*Give P values as exact values whenever suitable.* |
| ☒ | ☐ | For Bayesian analysis, information on the choice of priors and Markov chain Monte Carlo settings |
| ☒ | ☐ | For hierarchical and complex designs, identification of the appropriate level for tests and full reporting of outcomes |
| ☒ | ☐ | Estimates of effect sizes (e.g. Cohen's *d*, Pearson's *r*), indicating how they were calculated |

*Our web collection on statistics for biologists contains articles on many of the points above.*

## Software and code

Policy information about availability of computer code

| | |
|---|---|
| Data collection | CLARIOstar MARS 3.32; NMRPipe/NMRDraw (IBBR version) |
| Data analysis | Origin 2018, GraphPad Prism 7; NMRView J verion9.2.b20; Sparky (NMRFAM version) |

For manuscripts utilizing custom algorithms or software that are central to the research but not yet described in published literature, software must be made available to editors/reviewers. We strongly encourage code deposition in a community repository (e.g. GitHub). See the Nature Research guidelines for submitting code & software for further information.

## Data

Policy information about availability of data

All manuscripts must include a data availability statement. This statement should provide the following information, where applicable:

- Accession codes, unique identifiers, or web links for publicly available datasets
- A list of figures that have associated raw data
- A description of any restrictions on data availability

All the fluorescence raw data of displacement assay and affinity titration can be acquired from aeh49@duke.edu upon request. All the plaque assay, dual luciferase assay, and RNP-immunoprecipitation qPCR raw data can be acquired from brewerga@rwjms.rutgers.edu. All the NMR and calorimetry raw data can be acquired from bst18case.edu

# Field-specific reporting

Please select the one below that is the best fit for your research. If you are not sure, read the appropriate sections before making your selection.

☒ Life sciences ☐ Behavioural & social sciences ☐ Ecological, evolutionary & environmental sciences

For a reference copy of the document with all sections, see nature.com/documents/nr-reporting-summary-flat.pdf

# Life sciences study design

All studies must disclose on these points even when the disclosure is negative.

| | |
|---|---|
| Sample size | Serial dilutions from 1000-0.001 uM of the small molecules were used to test their antiviral activity. This is a 6-log10 range and was sufficient to identify small molecules with antiviral activity. For figures reporting statistics, N is at least 3. |
| Data exclusions | No data were excluded. |
| Replication | For all figures reporting statistics, N is at least 3. Otherwise, experimental repeat numbers are indicated in the relevant figure legends. |
| Randomization | This is not relevant to our study. The same cells were grown in multi-well plates for experiments. |
| Blinding | This is not relevant to our study. The same cells were grown in multi-well plates for experiments. |

# Reporting for specific materials, systems and methods

We require information from authors about some types of materials, experimental systems and methods used in many studies. Here, indicate whether each material, system or method listed is relevant to your study. If you are not sure if a list item applies to your research, read the appropriate section before selecting a response.

## Materials & experimental systems

| n/a | Involved in the study | |
|---|---|---|
| ☐ | ☒ | Antibodies |
| ☐ | ☒ | Eukaryotic cell lines |
| ☒ | ☐ | Palaeontology |
| ☒ | ☐ | Animals and other organisms |
| ☒ | ☐ | Human research participants |
| ☒ | ☐ | Clinical data |

## Methods

| n/a | Involved in the study | |
|---|---|---|
| ☒ | ☐ | ChIP-seq |
| ☒ | ☐ | Flow cytometry |
| ☒ | ☐ | MRI-based neuroimaging |

## Antibodies

| | |
|---|---|
| Antibodies used | Rabbit polyclonal antibody (serum) against AUF1; normal rabbit serum (as a non-immune control) |
| Validation | The AUF1 antibody was custom produced by Pocono Rabbit Farm (approx. 15 years ago) using purified AUF1 p37 isoform as immunogen. At that time, our laboratory performed Western blots of human cell extracts to verify detection of AUF1. Normal rabbit serum (non-immune control) was purchased from Sigma. |

## Eukaryotic cell lines

Policy information about cell lines

| | |
|---|---|
| Cell line source(s) | SF268 (human glioblastoma) was provided by a colleague; Vero (African green monkey kidney) was obtained from ATCC. |
| Authentication | STR analysis was performed by ATCC July 2020 for SF268; confirmed human origin. Cell line is a similar match to the STR profile for SF268 (RRID:CVCL_1689; https://web.expasy.org/cellosaurus) (data available upon request). ATCC performs STR analysis on human cells only. Thus, for Vero, they only provide the Product Information Sheet for the purchased batch (available upon request). |
| Mycoplasma contamination | Cell lines were not tested for mycoplasma contamination. |
| Commonly misidentified lines (See ICLAC register) | *Name any commonly misidentified cell lines used in the study and provide a rationale for their use.* |
