# Supplementary material for: IRES-targeting small molecule inhibits enterovirus 71 replication via allosteric stabilization of a ternary complex
Source: Nat Commun. 2020 Sep 22;11:4775. doi: 10.1038/s41467-020-18594-3 (PMC7508794; doi:10.1038/s41467-020-18594-3)
Supplement: Supplementary file 3 — Description of Additional Supplementary Files [file 41467_2020_18594_MOESM3_ESM.pdf]

Description of Additional Supplementary Files

File Name: Supplementary Movie 1
Description: Solution structure of the SLII-(DMA-135) complex. A morph video depicting the DMA-135 induced conformational change to SLII. The apo (PDB 5V17) and holo SLII (PDB 6XB7) structures are each rendered as a cartoon with a semitransparent surface. Residues colored red and shown as lines correspond to the binding surface for DMA-135. The morph reveals how DMA-135 changes the local stacking of the bulge loop to expose the AUF1 binding site.
